# Prebiotics as an adjunct therapy for posttraumatic stress disorder: a pilot randomized controlled trial

**DOI:** 10.3389/fnins.2024.1477519

**Published:** 2025-01-07

**Authors:** Robin M. Voigt, Phillip A. Engen, Michelle Villanueva, Simona A. Bambi, Stefan J. Green, Ankur Naqib, Shohreh Raeisi, Maliha Shaikh, Bruce R. Hamaker, Thaisa M. Cantu-Jungles, Sarah A. Pridgen, Philip Held, Ali Keshavarzian

**Affiliations:** ^1^Rush Center for Integrated Microbiome and Chronobiology Research, Rush University Medical Center, Chicago, IL, United States; ^2^Department of Internal Medicine, Rush University Medical Center, Chicago, IL, United States; ^3^Department of Anatomy and Cell Biology, Rush University Medical Center, Chicago, IL, United States; ^4^Genomics and Microbiome Core Facility, Rush University Medical Center, Chicago, IL, United States; ^5^Whistler Center for Carbohydrate Research, Department of Food Science, Purdue University, West Lafayette, IN, United States; ^6^Department of Psychiatry and Behavioral Science, Rush University Medical Center, Chicago, IL, United States; ^7^Department of Physiology, Rush University Medical Center, Chicago, IL, United States

**Keywords:** posttraumatic stress disorder (PTSD), gut-brain axis, prebiotics, microbiota modulation, veterans, microbiota, metabolites

## Abstract

**Introduction:**

Posttraumatic stress disorder (PTSD) is a debilitating disorder characterized by intrusive memories, avoidance, negative thoughts and moods, and heightened arousal. Many patients also report gastrointestinal symptoms. Cognitive behavioral therapy (CBT) is an evidence-based treatment approach for PTSD that successfully reduces symptoms. However, many patients still meet criteria for PTSD after treatment or continue to have symptoms indicating the need for new treatment strategies for PTSD. Patients with PTSD have a disrupted intestinal microbiome (i.e., dysbiosis) which can promote neuroinflammation; thus, modulation of the microbiome could be an alternative or adjunct treatment approach for PTSD.

**Methods:**

The current study was a 12-week, double-blind, placebo-controlled trial seeking to understand if CBT combined with a microbiota-modifying, prebiotic fiber intervention would beneficially impact clinical outcomes in veterans with PTSD (*n* = 70). This proof-of-concept, pilot trial was designed to assess: (1) the relationship between severity of PTSD symptoms and microbiota composition and SCFA levels (i.e., acetate, propionate, butyrate), (2) if CBT treatment with a concomitant prebiotic fiber intervention would beneficially impact clinical outcomes in veterans with PTSD, (3) evaluate the feasibility and acceptability of a prebiotic intervention as an adjunct treatment to CBT, and (4) assess the impact of treatment on the intestinal microbiota and stool SCFA (i.e., mechanism).

**Results:**

This study found that PTSD severity may be associated with reduced abundance of taxa capable of producing the SCFA propionate, and that a subset of individuals with PTSD may benefit from a microbiota-modifying prebiotic intervention.

**Conclusion:**

This study suggests that targeting the intestinal microbiome through prebiotic supplementation could represent a promising avenue for enhancing treatment outcomes in some individuals with PTSD.

**Clinical trial registration:**

https://clinicaltrials.gov/, identifier NCT05424146.

## Introduction

Posttraumatic stress disorder (PTSD) is a debilitating disorder that can develop following witnessing or experiencing a traumatic event. PTSD is characterized by reexperiencing traumatic events, avoidance of trauma-related stimuli, negative alterations in cognition or mood, and increased arousal and reactivity as well as dissociation in a subset. Additional gastrointestinal symptoms (e.g., pain/cramping, diarrhea, constipation, bloating/gas) can also manifest in individuals with PTSD. It is estimated that approximately 6–7% of the world population has PTSD with prevalence as high as 25–30% in combat-exposed veterans (Koenen et al., 2017; Fulton et al., 2015; Jordan et al., 1991; Tanielian et al., 2008).

Trauma-focused cognitive behavioral therapies (CBT), such as Cognitive Processing Therapy (CPT), are considered first-line interventions for the treatment of PTSD and successfully reduce PTSD symptoms (VA/Dod, 2017). Although CBT produces significant and clinically meaningful reductions in PTSD symptoms, many patients still meet criteria for PTSD after treatment or continue to have symptoms indicating the need for alternative or adjunct approaches to better treat veterans with PTSD (Steenkamp et al., 2015; Schottenbauer et al., 2008; Kovacevic et al., 2023). Modulation of the microbiome could be a new strategy to treat PTSD.

Studies demonstrate that PTSD is associated with an abnormal intestinal and oral microbiomes compared to controls without PTSD (Hemmings et al., 2017; Bajaj et al., 2019; Malan-Muller et al., 2022; Yoo et al., 2023; Zeamer et al., 2023; Levert-Levitt et al., 2022). These studies are inconsistent in terms of changes in the abundance of specific bacteria (which could reflect differences between study populations, diagnostic criteria for PTSD, medication use, co-morbid conditions, among other factors), but they consistently report that PTSD is associated with a proinflammatory stool microbiome and lower levels of beneficial metabolites produced by the microbiota including short chain fatty acids (SCFA) (Voigt et al., 2022). A proinflammatory microbiome promotes depression and anxiety and low levels of SCFA can promote inflammation (Chandel et al., 2024; Ferrari et al., 2024; Yao et al., 2022; Ney et al., 2023); therefore, it is not surprising that microbiota modulation has been proposed as an approach to treat PTSD.

Modulation of microbiota in patients with PTSD has been evaluated in a limited number of studies. The probiotic species *Lactobacillus rhamnosus* GG has anti-inflammatory effects and decreases stress-induced changes in heart rate in veterans with PTSD and co-occurring traumatic brain injury (Brenner et al., 2020; Brenner et al., 2022) and consumption of a fermented soy formulation (which can alter the intestinal microbiome) reduces symptoms (assessed via CAPS) in treatment-resistant veterans with PTSD (Gocan et al., 2012). Another approach to modify the intestinal microbiome is via consumption of prebiotic fibers which beneficially influence microbiota composition and increase SCFA levels (Gibson et al., 2017). To date, no studies have evaluated the utility of prebiotic fibers for the treatment of PTSD. This double-blind, randomized controlled, pilot trial administered CPT plus a microbiota-modifying prebiotic fiber or placebo intervention to determine: (1) the relationship between severity of PTSD symptoms and microbiota composition and SCFA levels (i.e., acetate, propionate, butyrate), (2) if CBT treatment with a concomitant prebiotic fiber intervention would beneficially impact clinical outcomes in veterans with PTSD, (3) evaluate the feasibility and acceptability of a prebiotic intervention as an adjunct treatment to CBT, and (4) assess the impact of treatment on the intestinal microbiota and stool SCFA (i.e., mechanism).

## Materials and methods

All research activities were approved by the Rush University Medical Center Institutional Review Board (IRB) (ORA 21051205) and the study was registered with ClinicalTrials.gov (Identifier NCT05424146). All participants provided written informed consent prior to participation.

### Study design

**FIGURE 1:**
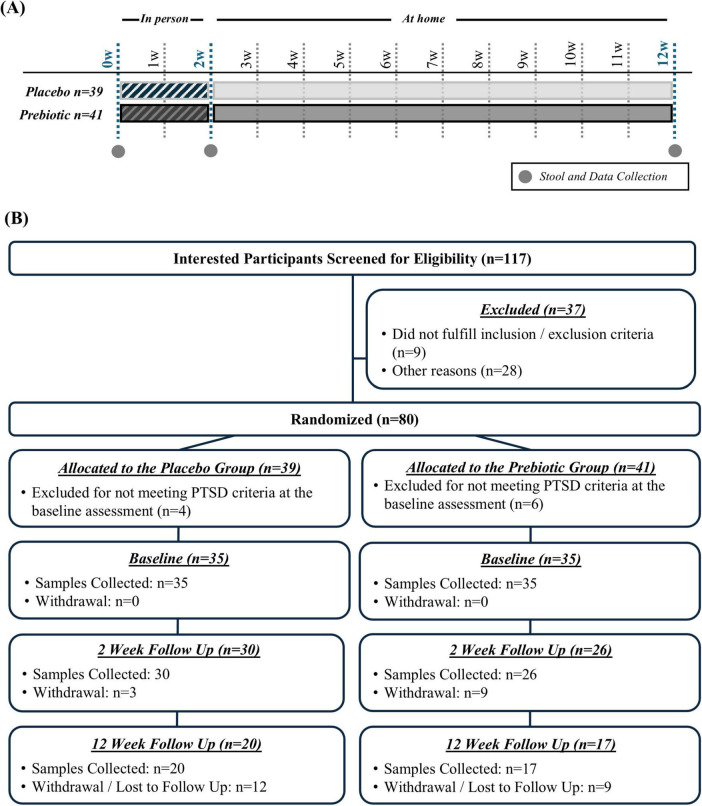
**(A)** Study design and **(B)** enrollment and retention.

This pilot study was a 12-week, double-blind, placebo-controlled trial in veterans with PTSD comparing the effects of a placebo versus a prebiotic fiber intervention with concurrent CBT (Figure 1A). The 12-week duration (i.e., 84 days) was based on the following criteria: (1) a prior study from our group demonstrates that the prebiotic formulation robustly changes the microbiota composition of Parkinson’s disease patients within 10 days (Hall et al., 2023) leaving more than 70 days for microbiota-induced changes to occur in this study and (2) many evidence-based PTSD therapies are designed to be delivered in 12 to 16 weekly sessions, roughly translating to 3–4 months of treatment. Participants who attended a 2-week CPT-based intensive PTSD treatment program (ITP) at The Road Home Program: National Center of Excellence for Veterans and Their Families at Rush University Medical Center (RHP, Chicago, IL) between June 2022 and March 2023 were offered the opportunity to participate in this study. As part of the ITP, participants receive 16 twice daily 50-min sessions of individual CPT as well as adjunctive services such as psychoeducation, art therapy, and mindfulness training. Veterans were assigned to either co-ed combat trauma or military sexual trauma cohorts depending on their index trauma. The program produces large and lasting PTSD symptom reduction (Held et al., 2023; Held et al., 2024). Additional information about the treatment program can be found in a recent publication (Held et al., 2023).

***Clinical Assessments***: Psychological assessments for PTSD were conducted as part of standard clinical procedures at: (1) baseline (BL, assessing past week PTSD symptoms), (2) day 10 of CBT following all clinical interventions (2-weeks (w), assessing past week PTSD symptoms), and (3) 12 weeks after CBT completion (12w, assessing past month PTSD symptoms). The PTSD Checklist for DSM-5 (PCL-5) (Weathers et al., 2018) ranges from 0 to 80 with higher scores indicating more severe PTSD symptoms.

***Other Assessments***: At baseline, participants completed the Diet History Questionnaire III (DHQ III), a dietary assessment tool designed to capture dietary habits over the prior month. The DHQ III assessment was conducted to exclude participants who were consuming a non-traditional diet (e.g., Paleo, Vegetarian). At the conclusion of the study, a post study survey was given to participants to assess the intervention including satisfaction, portion size, taste, texture, impact on appetite, and if they would continue the prebiotic or placebo intervention (Supplementary Table 1). Participants completed the NIH Patient-Reported Outcomes Measurement Information System (PROMIS GI) questionnaire to assess gastrointestinal symptoms.

***Biological Sample Collection***: Stool samples were collected: (1) at baseline (i.e., within the first 5 days of CPT), (2) 2w (i.e., between days 7 and 14), and (3) 12w (i.e., 10w after completing CPT). If a participant was not able to provide a baseline sample (i.e., within 5 days of starting CPT) they were excluded from the study. The prebiotic or placebo intervention was initiated after participants provided the baseline sample. Baseline and the 2w sample were obtained while participants were at the RHP and the 12w sample was collected at home. A kit to collect the 12w sample was sent to participants, which was returned to the laboratory via mail. Stool samples were collected using OMNIgene Gut (OMR-200; gut microbiome profiling) and OMNImet GUT (ME-200; gut metabolome profiling) collection kits with analysis at each collection time point (DNAGenotek, Kanata, Canada). These commercially available collection kits are a reliable way to collect and preserve stool samples at ambient temperature, making them ideal for home-based collection and transport to the lab (e.g., shipping). Once in the lab samples were aliquoted and stored at −80°C until analysis.

### Participants

One hundred seventeen interested participants were screened for eligibility, 37 were excluded because they did not meet inclusion/exclusion criteria or for other reasons (e.g., not interested in participation), and 80 participants were randomized (*n* = 39 placebo group, *n* = 41 prebiotic group) (Figure 1B).

***Inclusion Criteria***: (1) Veterans participating in the 2w ITP with a diagnosis of PTSD (PTSD diagnosis verified by clinicians at intake) and (2) the ability and willingness to consume two dietary supplement bars daily and collect stool.

***Exclusion Criteria***: (1) Active suicidality or homicidality, current engagement in non-suicidal self-harm, unmanaged mania or psychosis, current eating disorder, substance use which may lead to medical intervention if discontinued, (2) gastrointestinal disease: (a) intestinal resection, (b) history of GI disease (except hiatal hernia, non-proton pump inhibitor requiring gastroesophageal reflux disease, hemorrhoids), (c) known renal disease or liver dysfunction, (3) antibiotic use within the 12w prior to enrollment, (4) plan to have a major change in diet during the study, (5) allergy to almond, flax seed, or coconut.

The population was 54% female, predominately non-Hispanic (84%) white (67%), and the average age was 44 (range: 25–67). The population had 54% combat trauma and 46% military sexual trauma. Participant demographics are summarized in Table 1 and detailed in Supplementary Data Sheet 1.

**TABLE 1:** Participant demographics.

	Total cohort	Placebo group	Prebiotic group
**Number (n)**	70	35	35
**Age (mean ± SEM, range)**	44.2 ± 1.2 (25–67)	46.5 ± 1.9 (25–67)	42.0 ± 1.5 (28–62)
**Sex**			
Female (*n*, %)	38 (54.3%)	19 (54.3%)	19 (54.3%)
Male (*n*, %)	32 (45.7%)	16 (45.7%)	16 (45.7%)
**Race**			
Asian	4 (5.7%)	3 (8.6%)	1 (2.9%)
Black or African American	12 (17.1%)	9 (25.7%)	3 (8.6%)
Native Hawaiian or Pacific Islander	1 (1.4%)	0 (0.0%)	1 (2.9%)
Other	6 (8.6%)	3 (8.6%)	3 (8.6%)
White	47 (67.1%)	20 (57.1%)	27 (77.1%)
**Ethnicity**			
Not Hispanic or Latino	59 (84.3%)	28 (80.0%)	31 (88.6%)
Hispanic or Latino	11 (15.7%)	7 (20.0%)	4 (11.4%)
**Military Service Status**			
Active Duty	9 (12.9%)	4 (11.4%)	5 (14.3%)
Discharged	36 (51.4%)	17 (48.6%)	19 (54.3%)
Inactive Ready Reserve	1 (1.4%)	0 (0.0%)	1 (2.9%)
Medically Retired	10 (14.3%)	5 (14.3%)	5 (14.3%)
Reserves	4 (5.7%)	1 (2.9%)	3 (8.6%)
Retired	8 (11.4%)	6 (17.1%)	2 (5.7%)
Not Indicated	2 (2.9%)	2 (5.7%)	0 (0.0%)
**Cohort Type**			
Combat	38 (54.3%)	18 (51.4%)	20 (57.1%)
Military Sexual Trauma	32 (45.7%)	17 (48.6%)	15 (42.9%)
**PCL-5 (0–80) mean ± SEM, range, n)**			
Baseline	56.2 ± 1.5 (32–80) (*n* = 70)	56.4 ± 1.7 (35–74) (*n* = 35)	56.0 ± 2.4 (32–80) (*n* = 35)
2w	35.4 ± 2.1 (2–76) (*n* = 70)	37.6 ± 3.2 (2–76) (*n* = 35)	33.3 ± 2.8 (7–69) (*n* = 34)
12w	39.2 ± 2.5 (5–74) (*n* = 48)	40.0 ± 4.2 (5–74) (*n* = 22)	38.5 ± 3.0 (6–69) (*n* = 26)

### Intervention

All veterans in this study received the same kind of psychological intervention (i.e., CPT + adjunctive services) through the 2w ITP with either a prebiotic or placebo intervention. The prebiotic and placebo bars were identical except the inclusion of four prebiotic fibers (i.e., resistant starch, rice bran, resistant maltodextrin, inulin; 10g fiber/bar). The prebiotic fiber mixture was designed to promote complementary groups of core beneficial intestinal bacteria and augment production of all three SCFA (acetate, propionate, butyrate) (Hall et al., 2023). One bar was consumed daily during the first week and then two bars daily for the remaining 11 weeks (total of 161 bars). The prebiotic mixture is a proprietary formula (comprised of inulin, resistant starch type 2, resistant maltodextrin, and rice bran) developed by RiteCarbs LLC based on *ex vivo* stool fermentation studies as well as studies in humans demonstrating that this fiber mixture increases the abundance of bacteria that can produce SCFA, reduces the abundance of pro-inflammatory, Gram-negative bacteria, increases levels of SCFA, and reduces inflammation (Hall et al., 2023). The bars were produced by a licensed manufacturer and packaging company, Pure Bliss Organics. Ingredients of the bar were organic, and generally recognized as safe (GRAS), food-grade ingredients. The placebo bar was produced by the same manufacturer and used the same ingredients but without the prebiotic fibers and had identical packaging. During the 2w ITP, participants were provided healthy breakfast, lunch, and dinner options meals by Blue Plate, a catering company.

***Randomization***: Participants were randomized to placebo or prebiotic using a stratified randomization scheme which balanced the sample on demographic characteristics (sex, age) and baseline PTSD symptom severity (PCL-5 score) to ensure the active and control groups were balanced.

### Stool short-chain fatty acid metabolomics analysis

Stool samples collected using the OMNImet tube were dehydrated and approximately 50 mg of dry stool was placed into a 2-mL plastic tube, 0.2 mL of ethanol was added, the sample was vortexed for 15s, and then centrifuged at 3000 g × 10 min at 4°C. Then 40 μL of supernatant was transferred to a 2-mL glass vial and the samples were processed. Briefly; 20 μL of cold internal standard solution containing 10 mg/mL of 13C2-soidum acetate and 1 mg/mL of 13C4-sodium butyrate in ethanol, and 20 μL of 4 N NaOH were added to the samples. After vortexing, samples were dried under nitrogen. The dried extracts were added with 0.1 mL of 1 N HCl and 0.3 mL of cold MTBE and then vortexed for 15 s. About 80 μL of the top methyl tert-butyl ether (MTBE) layer were recovered after centrifugation at 3,000 g x 10 min at 4°C and stored at −20°C until SCFA analysis. A pooled quality control (QC) was generated by combining 30 μL of MTBE extract of each study sample.

The MTBE extract (1 μL) of SCFAs were injected into a Trace 1310 GC coupled to a Thermo ISQ-LT MS, at a 5:1 split ratio. The inlet was held at 240°C. SCFA separation was achieved on a 30m DB-WAXUI column (J&W, 0.25 mm ID, 0.25 μm film thickness). Oven temperature was held at 100°C for 0.5 min, ramped at 10°C/min to 175°C, then ramped to 240°C at 40°C/min, and held at 240°C for 3 min. Helium carrier gas flow was held at 1.2 mL/min. Temperatures of transfer line and ion source were both held at 250°C. SIM mode was used to scan ions 45, 60, 62, 73, 74, 88 at a rate of 10 scans/sec under electron impact mode. The injection order of samples was again randomized. Injector liners were replaced after every 60 samples. Calibration curves were analyzed after every six samples. Samples were split into three acquisition batches. Injector liner was replaced after each acquisition batch and calibration curve was acquired with each acquisition batch.

Gas chromatography-mass spectrometry data was processed using Chromeleon 7.2.10 software (Thermo Scientific). Peak areas were extracted for target compounds detected in biological samples and normalized to the peak area of the appropriate internal standard or surrogate in each sample.

### DNA extraction and next-generation sequencing

Automated DNA extraction of the stool samples was performed using a chemagic 360 instrument (Revvity, Shelton, CT, USA) with a chemagic DNA Stool 200 Kit H96 per manufacturer instructions. Stool samples were subject to bead-beating using a TissueLyser II device prior to purification on the chemagic instrument. Genomic DNA was PCR amplified with primers targeting the V4 variable region of microbial 16S rRNA genes using a two-stage PCR protocol, as described previously (Naqib et al., 2018). The primers contained 5′ common sequence tags known as Fluidigm common sequences 1 and 2 (CS1 and CS2). Primers CS1_515F and CS2_806R (modified from the primer set employed by the Earth Microbiome Project (EMP; ACAC TGACGACATGGTTCTACAGTGTGYCAGCMGCCGCGGTAA and TACGGTAGCAGAGACTTGGTCTCCGGACTACNVGGG TWTCTAAT, respectively – underlined regions represent linker sequences) were employed for the first stage amplifications. PCRs performed ten microliter reactions in 96-well plates, using repliQa HiFi ToughMix (Quantabio). PCR conditions were 98°C for 2 min, followed by 28 cycles of 98°C for 10 s, 52°C for 1 s and 68°C for 1 s.

Subsequently, a second PCR amplification was performed in ten microliter reactions in 96-well plates using the same PCR mastermix. Each well received a separate primer pair with a unique ten-base barcode, obtained from the Access Array Barcode Library for Illumina (Fluidigm, South San Francisco, CA; Item# 100-4876). One microliter of PCR product from the first stage amplification was used as template for the second stage, without cleanup. Cycling conditions were 98°C for 2 min, followed by eight cycles of 98°C for 10 s, 60°C for 1 s and 68°C for 1 s. Libraries pooled and sequenced with a 10% phiX spike-in on an Illumina Miniseq sequencer employing a mid-output flow cell (2 × 154 paired-end reads). Library preparation, pooling, and sequencing performed at the Genomics and Microbiome Core Facility (GMCF) at Rush University.

All laboratory analyses were conducted by staff blind to group assignment.

### Bioinformatics analysis of amplicon sequences

Microbiome bioinformatics were performed with the software package QIIME2 (version 2021.11) (Bolyen et al., 2019). Raw sequence data were checked for quality using FastQC and merged using PEAR (Zhang et al., 2014). Merged sequences were quality filtered using the q2-demux plugin followed by denoising with DADA2 (via q2-dada2)(Callahan et al., 2016). Primer adapter sequences were removed using cutadapt algorithm (Kechin et al., 2017). Alpha-diversity metrics (Shannon index, Simpson’s index, observed features, and Pielou’s evenness) and beta-diversity metrics were calculated using q2-diversity after samples were rarefied to a depth of 30,000 sequences per sample. Taxonomy was assigned using the q2-feature-classifier classify-sklearn naïve Bayes taxonomy classifier against the SILVA 138 99% reference database (Bokulich et al., 2018; Quast et al., 2013). The contaminant removal software, *decontam* (Davis et al., 2018), did not detect any contaminants based on the prevalence of amplicon sequence variants (ASVs) in the reagent negative blank controls using default parameters.

To assess microbial community compositions, we conducted Permutational Multivariate Analysis of Variance (PERMANOVA) (Kelly et al., 2015) and Permutational Analysis of Multivariate Dispersions (PERMDISP), (Anderson, 2006) both derived from Aitchinson distance, using 9,999 permutations and corrected for multiple testing using the Benjamini-Hochberg (BH) method on ASV counts. Centroid-based Non-metric Multi-dimensional Scaling (NMDS) plots generated for all metadata groups using vegan package in R. These plots generated based on ASV counts rarefied at 30,000 sequences. Based on whether the comparison was paired or unpaired, either the Wilcoxon signed-rank test or Centered Log-ratio Kruskal Wallis (CLR-KW) algorithm were used to identify significantly differentially abundant features (i.e., individual taxa, functional gene/pathways) between participant’s baseline, 2w and 12w prebiotic or placebo interventions. These results were corrected using the BH method. Differences in the relative abundance of individual taxa and functional genes/pathways with relative abundance greater than 0.1% were assessed for significance. An inferred modeling approach, using curated 16S rRNA microbial relative abundances of the genus taxonomic level, allowed us to identify individual taxa and group them accordingly based on their known involvement with total SCFA-production and putative Gram-negative proinflammatory-production (Singh et al., 2024). Subgroup analyses of SCFA-producing, acetate-producing, butyrate-producing, propionate-producing, and Gram-negative bacterial taxa were evaluated. The subgroup analyses reflect a deliberate approach to explore nuanced differences in microbiota populations. First, understanding which taxa are enriched or depleted in PTSD patients could aid in the development of a prebiotic mixture that is targeted toward the specific aberrations observed in PTSD patients. Second, while the overall production of SCFA is a key indicator of prebiotic efficacy, it is crucial to recognize that individual SCFA may be differentially impacted by the prebiotic intervention as each have different physiological roles and are produced by different bacterial populations. Third, examining the abundance of Gram-negative bacteria is important to understand the utility of the intervention to modify the abundances of these pro-inflammatory bacteria which could underpin the sustain inflammation in individuals with PTSD. Bacteria that produce SCFA (i.e., acetate, butyrate, propionate) and Gram-negative bacteria were identified via literature search on PubMed (Louis and Flint, 2009; Vital et al., 2014; Vital et al., 2013; Flint et al., 2012; Basson et al., 2016; Louis et al., 2010; Rajilic-Stojanovic and De Vos, 2014; Hakansson and Molin, 2011; Chassaing and Darfeuille-Michaud, 2011; Louis et al., 2014; Kostic et al., 2014; Louis et al., 2004; Gevers et al., 2014; Lupp et al., 2007). A list of the specific bacterial genera under each umbrella (i.e., SCFA-producing and Gram negative) are detailed in Supplementary Data Sheet 2.

### Statistical analysis

Descriptive statistics, Student’s *t*-test, linear regression, or analysis of variance (ANOVA) were utilized as appropriate using GraphPad Prism 10.0 (GraphPad Software, San Diego, California, USA). For the two-way ANOVA analysis, a mixed effects model or repeated measures model was used with factors being intervention (i.e., placebo, prebiotic) and time (BL, 2w, 12w) followed by a *post hoc* Tukey test when a significant effect of time, intervention, or an interaction was observed. For subgroup analysis, participants were divided into males versus females. The analysis plan was intention-to-treat as it provides a realistic estimate of the effectiveness of a treatment in real-world settings, where adherence and compliance may not be perfect.

## Results

### PTSD severity at baseline associated with subtle differences in the intestinal microenvironment

**FIGURE 2:**
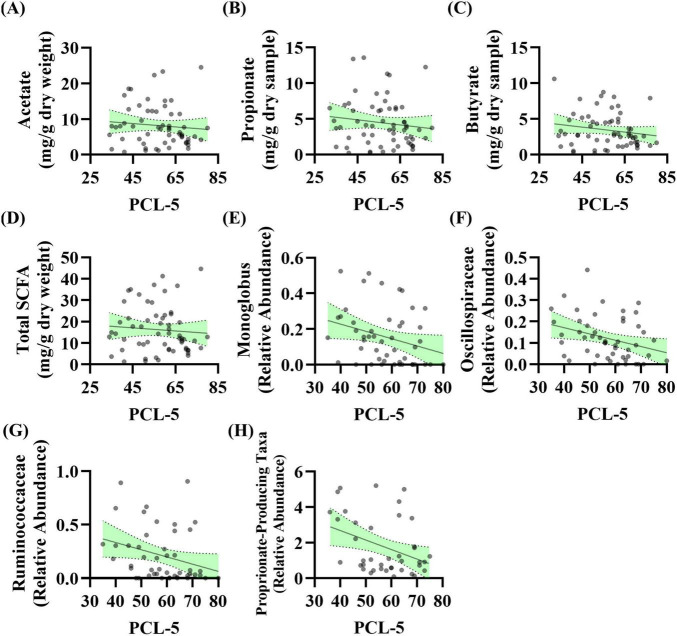
PTSD severity is associated with the abundance of taxa that produce short-chain fatty acids (SCFA) levels at baseline. **(A–D)** Linear regression analysis was conducted between baseline PCL-5 scores and baseline stool SCFA levels. No relationship observed for panel **(A)** acetate (*R*^2^ = 0.009, *F*_(1_, _57)_ = 0.544, *p* = 0.464, β = –0.047, *n* = 59), **(B)** propionate (*R*^2^ = 0.017, *F*_(1_, _59)_ = 1.014, *p* = 0.318, β = –0.035, *n* = 61), **(C)** butyrate (*R*^2^ = 0.031, *F*_(1_, _59)_ = 1.885, *p* = 0.175, β = –0.035, *n* = 61), or **(D)** total SCFA (*R*^2^ = 0.006, *F*_(1_, _58)_ = 0.366, *p* = 0.548, β = –0.072, *n* = 60). **(E–H)** Linear regression analysis was conducted between baseline PCL-5 score and baseline microbiota features (*n* = 53): **(E)** Genera *Monoglobus* (*R*^2^ = 0.083, *F*_(1_, _46)_ = 4.140, *p* = 0.048, β = –0.004, *n* = 48), **(F)** Oscillospiraceae Unclassified (*R*^2^ = 0.099, *F*_(1_, _48)_ = 5.280, *p* = 0.026, β = –0.003, *n* = 50), **(G)** Ruminococcaceae Unclassified (*R*^2^ = 0.083, *F*_(1_, _45)_ = 4.098, *p* = 0.049, β = –0.007, *n* = 47), **(H)** propionate-producing bacteria (*R*^2^ = 0.134, *F*_(1_, _38)_ = 5.896, *p* = 0.020, β = –0.054, *n* = 40). After correcting for the 68 taxonomic comparisons no q values were significant.

***Stool Short-Chain Fatty Acids*:** The relationship between baseline PCL-5 scores (i.e., PTSD symptoms) and stool SCFA levels was evaluated. Linear regression analysis revealed no significant relationship between acetate (*p* = 0.464), propionate (*p* = 0.318), butyrate (*p* = 0.175), or total SCFA levels (*p* = 0.548) with PCL-5 scores (Figures 2A–D). These data were not normally distributed; however, log transformation of the data did not impact results (data not shown).

***Stool Microbiota:*** The relationship between baseline PCL-5 scores (i.e., PTSD symptoms) and stool microbiota features was evaluated. Linear regression analysis showed that three genera as well as a curated list of bacteria that can produce propionate were significantly associated with PCL-5 score. The relative abundance of genera *Monoglobus* (*p* = 0.048), Oscillospiraceae Unclassified (*p* = 0.026), and Ruminococcaceae Unclassified (*p* = 0.049) were negatively associated with PCL-5 score (Figures 2E–G). Additionally, the relative abundance of a group of bacteria that can produce the SCFA propionate were negatively associated with PCL-5 score (*p* = 0.020, Figure 2H). After correcting for the 68 taxonomic comparisons no q values were significant (Supplementary Table 2).

### The prebiotic intervention beneficially impacts male veterans with PTSD

**FIGURE 3:**
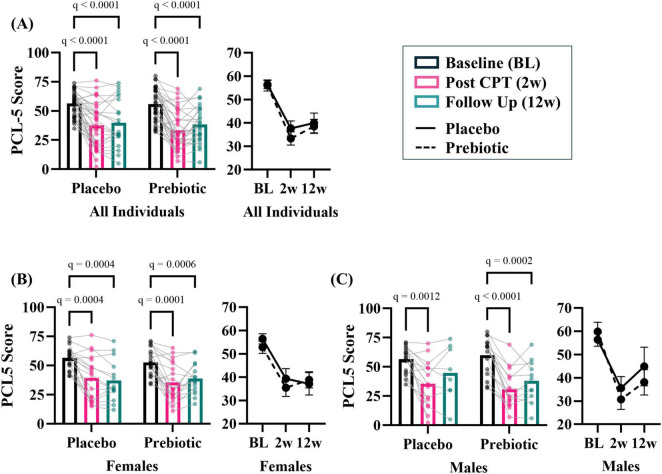
The prebiotic intervention impacts PCL-5 outcomes in a subset of participants. **(A)** There was a significant main effect of time but no impact of the prebiotic intervention (T: *p* < 0.001, *F*_(2_, _113)_ = 77.14; I: *p* = 0.653; *F*_(1_, _68)_ = 0.204; T x I: *p* = 0.491, *F*_(2_, _113)_ = 0.717), *post hoc* analysis indicating PCL-5 score decreased at 2w and 12w compared to baseline in both groups. Exploratory, subgroup analysis revealed that men may be more sensitive to the effects of the prebiotic intervention than females. **(B)** Females. There was a significant effect of time (T: *p* < 0.001, *F*_(1_._6_, _51_._7)_ = 39.500; I: *p* = 0.757, *F*_(1_, _36)_ = 0.097; T x I: *p* = 0.214, *F*_(2_, _63)_ = 1.580), and *post hoc* analysis revealed both placebo and prebiotic groups had significant reductions in PCL-5 score at 2w and 12w. **(C)** Males. There was a significant effect of time (T: *p* < 0.001, F_(1_._8_, _41_._7)_ = 40.420; I: *p* = 0.664, *F*_(1_, _30)_ = 0.192; T x I: *p* = 0.265, *F*_(2_, _46)_ = 1.367) and *post hoc* analysis revealed a significant decrease in PCL-5 score at 2w compared to the baseline in the placebo group whereas the prebiotic group PCL-5 was significantly reduced at both 2w and 12w compared to baseline. All individuals: *n* = 22–35/group; Females: *n* = 14–19/group; Males: *n* = 8–16/group. Average ± standard error of the mean. Two-way mixed model ANOVA (factors: time, intervention) with *post hoc* Tukey. T = time, I: intervention, T x I = interaction.

As a group, the prebiotic intervention did not impact PCL-5 score (i.e., PTSD symptom reduction). There was a significant effect of time (time: *p* < 0.001, intervention: *p* = 0.653, interaction: *p* = 0.491) with both placebo and prebiotic groups demonstrating a significant reduction in PCL-5 score compared to baseline (Figure 3A; BL vs 12w: Placebo group Cohen’s *d* = 1.117, Paired *t*-test: *t*_(21)_ = 4.88, *p* < 0.001, Prebiotic group Cohen’s *d* = 1.204, Paired *t*-test: *t*_(25)_ = 6.79, *p* < 0.001; Placebo 12w vs Prebiotic 12w: Cohen’s *d* = 0.083, Student’s *t*-test: *t*_(46)_ = 0.286, *p* = 0.776). It is important to note that analysis of all participants (including those with missing data, mixed model ANOVA) versus those participants with data from all three time points resulted in the same statistical conclusions (two-way repeated measures ANOVA, data not shown). Additionally, no statistical differences were noted between participants who dropped out versus those who were retained in the study (data not shown).

The prebiotic intervention is a microbiota-modifying intervention, therefore baseline stool beta-diversity microbial community was evaluated based on sex. Stool microbial communities were significantly different (PERMANOVA: *p* = 0.017), no differences in group dispersions were noted (PERMDISP: *p* = 0.821) (Supplementary Figure 1A) between females and males. Differentially abundant taxa were identified between sexes at baseline (Supplementary Figures 1B, C). Examination of curated microbial SCFA-producing taxa and Gram-negative taxa by sex did not reveal differences at baseline (Supplementary Figures 1D, E). Male and female baseline demographics are shown in Supplementary Figure 1F. These populations were statistically indistinguishable (*p* > 0.05) except for cohort type (i.e., females had higher military sexual trauma and males higher combat trauma). These data served as a strong rationale to evaluate sex differences in response to the prebiotic intervention.

Analyses suggest that men may be more sensitive to the effects of the prebiotic intervention than women. In females, there was a significant main effect of time (time: *p* < 0.001, intervention: *p* = 0.757, interaction: *p* = 0.214) and *post hoc* analysis showed that PCL-5 symptoms were significantly reduced at 2w and 12w in both the placebo and prebiotic groups (Figure 3B; Placebo group Cohen’s *d* = 1.405, Paired *t*-test: *t*_(13)_ = 4.88, *p* < 0.001, Prebiotic group Cohen’s *d* = 1.134, Paired *t*-test: *t*_(14)_ = 4.210, *p* = 0.001; Placebo 12w vs Prebiotic 12w: Cohen’s *d* = −0.105, Student’s *t*-test: *t*_(27)_ = 0.283, *p* = 0.779). These results contrast with what was observed in males. In males, there was also a significant main effect of the time (time: *p* < 0.01, intervention: *p* = 0.664, interaction: *p* = 0.265), but *post hoc* analysis revealed that the placebo group demonstrated a significant reduction in PCL-5 score at 2w, whereas the prebiotic group was significantly reduced at both 2w and 12w (Figure 3C; Placebo group Cohen’s *d* = 0.709, Paired *t*-test: *t*_(7)_ = 1.95, *p* = 0.093, Prebiotic group Cohen’s *d* = 1.284, Paired *t*-test: *t*_(10)_ = 6.65, *p* < 0.001; Placebo 12w vs Prebiotic 12w: Cohen’s *d* = 0.330, Student’s *t*-test: *t*_(17)_ = 0.710, *p* = 0.487). The results in females and males were recapitulated when only participants with data from all three time points were included (i.e., two-way repeated measures ANOVA, data not shown). Subject demographics based on sex shown in Supplementary Table 3.

### The prebiotic intervention was feasible and acceptable

After completing the study, participants were given a post study questionnaire to assess feasibility and acceptability of the prebiotic intervention. Forty-four participants completed the questionnaires (Supplementary Table 4). *Compliance*: The average number of bars consumed during the 12 weeks was 138 (out of 161) indicating an average compliance of 86% of bars consumed. Participants in the prebiotic group self-reported higher compliance (151/161, 94%) than those in the placebo group (129/161, 80%). *Feasibility*: When examining the willingness of participants to consume 1–3 bars per day (0 = not at all, 5 = moderately, 10 = very much) responses indicated that consuming one bar per day would be highly acceptable (8.07 ± 0.41), two bars per day (the “dose” in the current study) moderately acceptable (5.34 ± 0.60). *Acceptability*: When queried about the likelihood of continuing the intervention the response for the whole cohort was positive (6.48 ± 0.62) with the prebiotic cohort having a higher likelihood of continuing the intervention compared to those in the placebo group [7.14 ± 0.81 vs 5.87 ± 0.92, respectively, although this was not significant (Mann-Whitney test)] (0 = not at all, 5 = moderately, 10 = very much).

**FIGURE 4:**
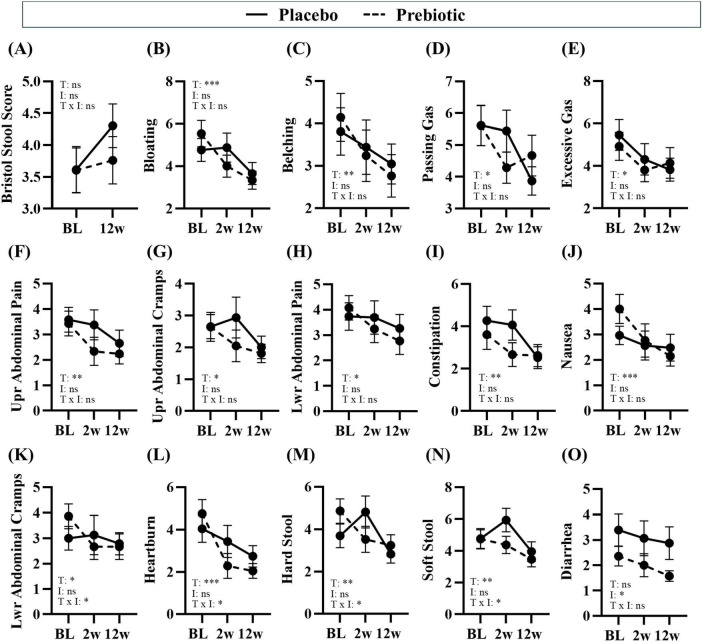
Treatment associated with improvements in gastrointestinal symptoms. Gastrointestinal symptoms were self-reported via the Bristol Stool Score and PROMIS GI questionnaire. **(A–E)** consumption of prebiotic fibers can induce gas and differences in stool consistency, but the prebiotic intervention did not induce negative side effects. **(B–N)** Time was a significant main effect for many gastrointestinal symptoms, **(K–N)** there was a significant main effect of the treatment or a time x treatment interaction for some outcomes, and **(O)** there was a main effect of the prebiotic intervention for one outcome. Placebo: *n* = 16–26/group; Prebiotic: *n* = 21–28/group. Average ± standard error of the mean. Two-way mixed model ANOVA (factors: time, intervention) with *post hoc* Tukey. *p < 0.05, **p < 0.01, ***p < 0.001 (see Supplementary Table 5 for details). T = time, I: intervention, T x I = interaction, ns = not significant. **(A)** T: *p* = 0.108, *F*_(1_, _38)_ = 2.707; I: *p* = 0.296, *F*_(1_, _56)_ = 1.114; T x I: *p* = 0.166, *F*_(1_, _38)_ = 1.994; **(B)** T: *p* = 0.002, *F*_(1_._7_, _60_._8)_ = 8.043; I: *p* = 0.720, *F*_(1_, _57)_ = 0.130; T x I: *p* = 0.130, *F*_(2_, _72)_ = 2.103; **(C)** T: *p* = 0.004, *F*_(1_._5_, _53_._7)_ = 7.096, I: *p* = 0.703, *F*_(1_, _57)_ = 0.147, T x I: *p* = 0.468, *F*_(2_, _72)_ = 0.768; **(D)** T: *p* = 0.029, *F*_(1_._7_, _59_._5)_ = 4.069; I: *p* = 0.948, *F*_(1_, _57)_ = 0.004; T x I: *p* = 0.151, *F*_(2_, _72)_ = 1.938; **(E)** T: *p* = 0.028, *F*_(1_._5_, _53_._0)_ = 4.363; I: *p* = 0.943, *F*_(1_, _57)_ = 0.005; T x I: *p* = 0.455, *F*_(2_, _72)_ = 0.796; **(F)** T: *p* = 0.003, *F*_(1_._5_, _53_._1)_ = 7.912; I: *p* = 0.342, *F*_(1_, _57)_ = 0.920; T x I: *p* = 0.367, *F*_(2_, _72)_ = 1.018; **(G)** T: *p* = 0.014, *F*_(1_._9_, _68_._2)_ = 4.705; I: *p* = 0.302, *F*_(1_, _57)_ = 1.086; T x I: *p* = 0.152, *F*_(2_, _72)_ = 1.935; **(H)** T: *p* = 0.026, *F*_(1_._4_, _49_._1)_ = 4.628; I: *p* = 0.473, *F*_(1_, _57)_ = 0.521; T x I: *p* = 0.161, *F*_(2_, _72)_ = 1.874; **(I)** T: *p* = 0.001, *F*_(2_._0_, _71_._1)_ = 8.333; I: *p* = 0.499, *F*_(1_, _57)_ = 0.463; T x I: *p* = 0.103, *F*_(2_, _72)_ = 2.349; **(J)** T: *p* < 0.001, *F*_(1_._9_, _67_._1)_ = 9.484, I: *p* = 0.861, *F*_(1_, _57)_ = 0.031; T x I: *p* = 0.060, *F*_(2_, _72)_ = 2.931; **(K)** T: *p* = 0.046, *F*_(1_._8_, _63_._6)_ = 3.380; I: *p* = 0.919, *F*_(1_, _57)_ = 0.010; T x I: *p* = 0.043, *F*_(2_, _72)_ = 3.297; **(L)** T: *p* < 0.001, *F*_(1_._7_, _61_._2)_ = 14.310, I: *p* = 0.672, *F*_(1_, _57)_ = 0.181; T x I: *p* = 0.041, *F*_(2_, _72)_ = 3.349; **(M)** T: *p* = 0.002, *F*_(2_._0_, _72_._0)_ = 7.028; I: *p* = 0.477, *F*_(1_, _57)_ = 0.512; T x I: *p* = 0.017, *F*_(2_, _72)_ = 4.306; **(N)** T: *p* = 0.009, F_(1_._9_, _67_._4)_ = 5.260; I: *p* = 0.281, *F*_(1_, _57)_ = 1.183; T x I: *p* = 0.021, *F*_(2_, _72)_ = 4.085; **(O)** T: *p* = 0.161, *F*_(1_._8_, _65_._3)_ = 1.904; I: *p* = 0.040, *F*_(1_, _57)_ = 4.401; T x I: *p* = 0.298, *F*_(2_, _72)_ = 1.233.

Consumption of prebiotic fiber can sometimes be associated with negative side effects such as gas and bloating; however, no negative side effects on gastrointestinal function were self-reported via questionnaire (i.e., Bristol Stool Scale and PROMIS GI) (Figure 4). However, four individuals who dropped out of the study reported reasons such as the bars were too filling, stomach upset/cramps, and bloating which were observed in both the placebo and prebiotic intervention groups.

### Treatment-induced changes in the intestinal micro-environment

The mechanism of the prebiotic-induced effects was hypothesized to be due to beneficial changes in the intestinal microenvironment; thus, the impact on gastrointestinal function, stool microbiota, and stool SCFA were assessed.

#### Gastrointestinal effects

Numerous beneficial changes in gastrointestinal function were noted over the 12w study in both the placebo and prebiotic groups. Specifically, significant reductions in abdominal pain and cramping, bloating/gas, heartburn/indigestion, nausea, were reported as well as improved constipation/regularity, stool consistency, and more satisfying bowel movements (i.e., less straining, sensation of complete emptying) (main effect of time: *p* < 0.05, Figures 4F–N) were reported in both groups. Several outcomes demonstrated a significant time x intervention interaction including: less lower abdominal cramping, improvements in stool consistency (i.e., less hard stool and less soft stool), and reduced heartburn (time x intervention interaction: *p* < 0.05, Figures 4K–N). One outcome demonstrated a significant main effect of the intervention wherein diarrhea was significantly reduced by the prebiotic intervention (main effect of the prebiotic intervention: *p* < 0.05, Figure 4O), which is likely driven by the smaller variance in the prebiotic intervention group. Statistical details are in Supplementary Table 5.

#### Stool short-chain fatty acids

**FIGURE 5:**
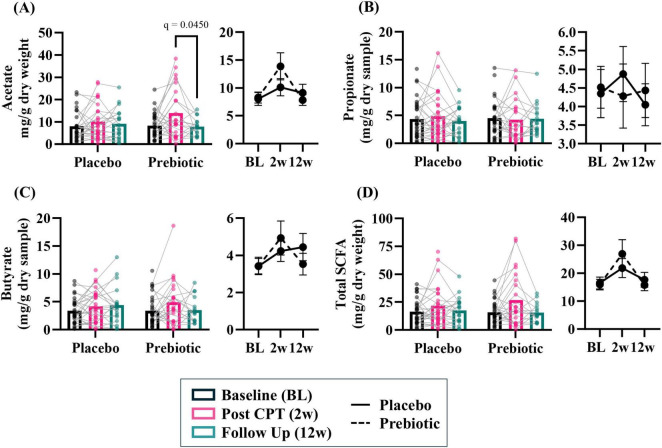
The prebiotic intervention associated with a transient increase in stool short chain fatty acid (SCFA) levels. A significant effect of time was noted for several SCFA, but no significant main effects of the intervention nor an interaction were noted: **(A)** acetate (T: *p* = 0.017, *F*_(1_._9_, _72_._4)_ = 4.475; I: *p* = 0.734, *F*_(1_, _61)_ = 0.117; T x I: *p* = 0.148, *F*_(2_, _78)_ = 1.959), **(B)** propionate (T: *p* = 0.896, *F*_(1_._8_, _67_._8)_ = 0.089, I: *p* = 0.855, *F*_(1_, _63)_ = 0.034; T x I: *p* = 0.888, *F*_(2_, _76)_ = 0.119), **(C)** butyrate (T: *p* = 0.051, *F*_(1_._7_, _65_._8)_ = 3.268; I: *p* = 0.917, *F*_(1_, _63)_ = 0.011; T x I: *p* = 0.493, *F*_(2_, _76)_ = 0.715), **(D)** total SCFA (i.e., acetate + propionate + butyrate; T: *p* = 0.010, *F*_(1_._7_, _67_._9)_ = 5.277; I: *p* = 0.988, *F*_(1_, _62)_ < 0.001; T x I: *p* = 0.309, *F*_(2_, _79)_ = 1.193). Outliers from each group were omitted prior to analysis. Placebo: *n* = 20–30/group; Prebiotic: *n* = 16–31/group. Average ± standard error of the mean. Two-way mixed model ANOVA (factors: time, intervention) with *post hoc* Tukey. T = time, I: intervention, T x I = interaction.

The prebiotic intervention-induced a transient increase in SCFA levels. Acetate demonstrated a significant impact of the time (time: *p* = 0.017, intervention: *p* = 0.734, interaction: *p* = 0.148) and *post hoc* analysis showed that in the prebiotic group levels of acetate were higher at 2w (*p* = 0.045) compared to 12w (Figure 5A). No effects were observed for propionate (time: *p* = 0.900, intervention: *p* = 0.856, interaction: *p* = 0.888) or butyrate (time: *p* = 0.051, intervention: *p* = 0.917, interaction: *p* = 0.493) (Figures 5B, C). Analysis of total SCFA showed a significant impact of time (time: *p* = 0.010, intervention: *p* = 0.988, interaction: *p* = 0.309) but no *post hoc* differences were noted (Figure 5D).

Analysis of SCFA levels based on sex revealed that stool SCFA were more impacted in males than females. There were no significant main effects observed in females (Supplementary Figures 2A–D, all *p* > 0.05). However, males exhibited a significant effect of time for acetate (time: *p* = 0.005, intervention: *p* = 0.538, interaction: *p* = 0.242), propionate (time: *p* = 0.038, intervention: *p* = 0.226, interaction: *p* = 0.285), and butyrate (time: *p* = 0.035, intervention: 0.917, interaction: *p* = 0.858) (Supplementary Figures 2E–G). Short chain fatty acid data are provided as raw data in Supplementary Data Sheet 1.

#### Stool microbiota

**FIGURE 6:**
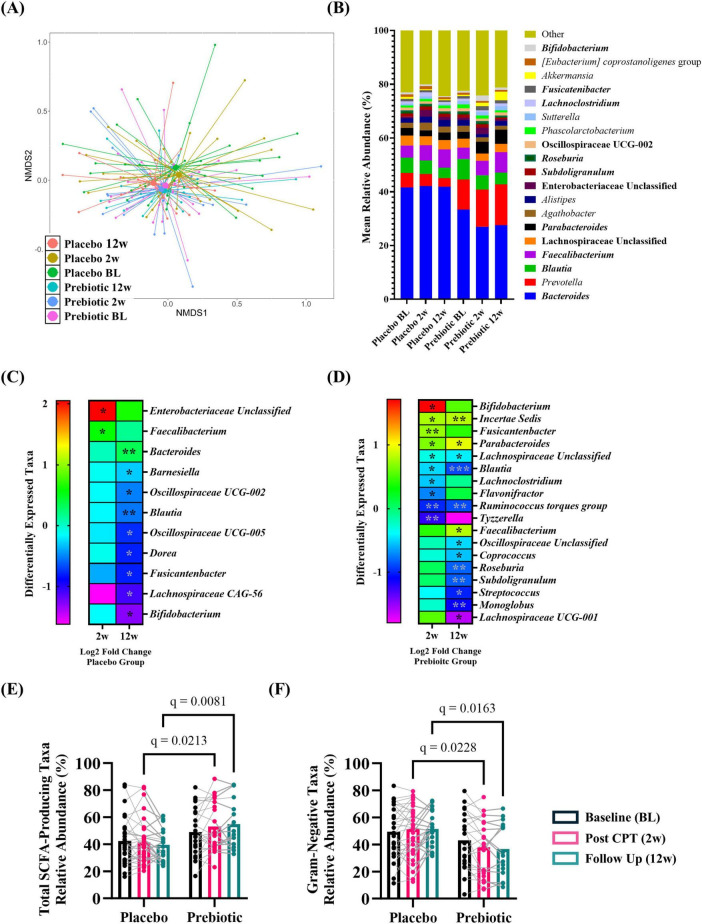
The prebiotic intervention associated with taxonomic differences in the stool microbiota community. When evaluated as a group, there were **(A)** no differences in overall microbial community structure. **(B)** Stacked histograms display the mean relative abundance of microbial genera [bold, *q* < 0.001 (corrected for multiple comparisons)]. Analysis of differentially abundant taxa as log2 fold change from baseline revealed differences in specific genera in **(C)** placebo and **(D)** prebiotic groups [Wilcoxon signed-rank test: *q < 0.05, **q < 0.01, ***q < 0.001 (corrected for multiple comparisons)]. Analysis of curated lists of genera revealed: **(E)** SCFA-producing taxa: A significant effect of the intervention and an interaction (T: *p* = 0.260, *F*_(1_._9_, _80_._8)_ = 1.370, I: *p* = 0.005, *F*_(1_, _54)_ = 8.570; T x I: *p* = 0.009, *F*_(2_, _84)_ = 4.986), with *post hoc* testing indicating SCFA-producing taxa were significantly higher in the prebiotic group at 2w and 12w compared to the placebo group. **(F)** Gram-negative taxa: A significant effect of intervention and an interaction were noted (T: *p* = 0.119, *F*_(1_._9_, _79_._0)_ = 2.214; I: *p* = 0.009, *F*_(1_, _54)_ = 7.311, T x I: *p* = 0.002, *F*_(2_, _84)_ = 6.656), as *post hoc* testing indicated lower abundances of Gram-negative taxa in the prebiotic group at 2w and 12w compared to the prebiotic group, as well as a significant reduction in the prebiotic group across time (baseline vs 12w). **(A,B)** (PERMANOVA/PERDISP: Supplementary Data Sheet 1; Centroid based NMDS plot, Aitchinson distance). **(C,D)** Wilcoxon-signed rank test: Supplementary Data Sheet 2; **(E,F)** mixed Model ANOVA with *post hoc* Tukey.

Analysis of microbial alpha-diversity indices (Supplementary Figures 3A–D, all *p* > 0.05) and beta-diversity stool microbial community structures (Figure 6A; PERMANOVA/PERMDISP, *p* > 0.05) revealed no significant effects. However, between group differences in specific taxa were observed (Figure 6B, bold taxa: *q* < 0.05, corrected for multiple comparisons). Microbiota communities are highly variable, thus evaluating changes across time relative to the baseline is a powerful approach to evaluate treatment-induced effects. This analysis approach revealed the placebo group had significantly increased relative abundance of genera Enterobacteriaceae Unclassified (2w), *Faecalibacterium* (2w), and *Bacteroides* (12w) as well as decreased relative abundance of numerous genera at 12w including *Blautia*, *Dorea*, and *Bifidobacterium* [Figure 6C (corrected for multiple comparisons)]. A greater number of differentially abundant taxa were observed in the prebiotic group. At 2w there was increased relative abundance of genera *Bifidobacterium*, *Incertae Sedis*, *Fusicantenbacter*, and *Parabacteroides* as well as a significant decrease in the relative abundance of genera Lachnospiraceae Unclassified, *Blautia*, *Lachnoclostridium*, *Flavonifactor*, *Ruminococcus torques* group, and *Tyzzerella*. Several of these alterations were maintained at 12w including *Incertae Sedis*, *Parabacteroides*, Lachnospiraceae Unclassified, *Blautia*, and *Ruminococcus torques* group. Additional differentially abundant taxa emerged at 12w including an increased relative abundance of genus *Faecalibacterium* and decreased relative abundances of genera Oscillospiraceae Unclassified, *Coprococcus*, *Roseburia*, *Subdoligranulum*, *Streptococcus*, *Monoglobus*, and *Lachnospiraceae UCG-001* [Figure 6D (corrected for multiple comparisons)]. Both the placebo and prebiotic groups exhibited a significant reduction in the relative abundance of the genus *Blautia* as well as different genera derived from taxonomic families of Lachnospiraceae and Oscillospiraceae at 12w. A discordant change of note is *Bifidobacterium* which decreased in the placebo group (12w) and increased in the prebiotic group (2w).

Next, the relative abundance of putative beneficial SCFA-producing genera and Gram-negative proinflammatory genera were evaluated. Analysis of SCFA-producing genera revealed significant main effects of intervention and a time x intervention interaction (time: *p* = 0.260, intervention: *p* = 0.005, interaction: *p* = 0.009) with *post hoc* analysis identifying that the relative abundance of SCFA-producing taxa was significantly higher in the prebiotic group compared to the placebo group at 2w and 12w (Figure 6E). Analysis of Gram-negative, proinflammatory bacteria revealed a significant main effect of the intervention and a time x intervention interaction (time: *p* = 0.119, intervention, *p* = 0.009, interaction: *p* = 0.002) with *post hoc* analysis identifying lower relative abundance of Gram-negative bacteria in the prebiotic group compared to the placebo group at 2w and 12w (Figure 6F).

Next, intervention induced changes were evaluated based on sex. It is important to note that males and females consumed similar levels of fiber at baseline (insoluble fiber: *t*_(34)_ = 0.897, *p* = 0.376; soluble fiber: *t*_(34)_ = 1.257, *p* = 0.217; total fiber: *t*_(34)_ = 1.085, *p* = 0.286). The male microbiome was more sensitive to the effects of the intervention than females. *Females*: Microbial alpha-diversity indices (Supplementary Figures 3E–H, all *p* > 0.05) and beta-diversity microbial community structures (Supplementary Figure 4A; PERMANOVA/PERMDISP, *p* > 0.05) revealed no significant differences. However, between group differences in specific taxa were observed [Supplementary Figure 4B, bold taxa: *q* < 0.05 (corrected for multiple comparisons)]. Evaluating changes across time relative to the baseline revealed that females receiving the placebo intervention had a significant increase in the relative abundance of genera *Phascolarctobacterium* at 2w and *Bacteroides* at 12w as well as a decrease in *Incertae Sedis* at 2w. In the prebiotic group, the relative abundance of the genus *Sutterella* was significantly increased at 2w and nine different taxa exhibited significantly reduced relative abundance at 2w and 12w including *Blautia, Roseburia*, several Clostridia-associated genera, among others. *Incertae Sedis* was altered in both the placebo and prebiotic groups which decreased in the placebo group (2w) and increased in the prebiotic group (2w, 12w) (Supplementary Figures 4C, D). Evaluation of a curated list of SCFA-producing bacteria revealed a significant main effect of the intervention (time: *p* = 0.254, intervention: *p* = 0.025, interaction: *p* = 0.252) but no *post hoc* differences were identified (Supplementary Figure 4E). There was a significant main effect of the intervention on the relative abundance of a curated list of Gram-negative bacteria (time: *p* = 0.164, intervention: *p* = 0.044, interaction: *p* = 0.170) but no *post hoc* differences were noted (Supplementary Figure 4F). *Males*: Microbial alpha-diversity indices (Supplementary Figures 3I–L, all *p* > 0.05) and beta-diversity stool microbial community structures (Supplementary Figure 5A; PERMANOVA/PERMDISP, all *p* > 0.05) revealed no differences. However, there were specific bacterial taxa that were differentially altered at the taxonomic level of genus [Supplementary Figure 5B, bold taxa: *q* < 0.05 (corrected for multiple comparisons)]. Evaluating changes across time revealed that males in the placebo group had a significant increase in the relative abundance of genera *Parasutterella* and *Bilophilia*, as well as a decrease in nine different genera at 12w including *Blautia, Dorea*, and *Bifidobacterium*. The prebiotic group exhibited a significant increase in the relative abundance of *Bifidobacterium* (2w, 12w), *Parabacteroides* (2w, 12w), *Anaerostipes* (2w), *Fusicatenibacter* (2w) and *Faecalibacterium* (12w). The abundance of numerous genera was significantly decreased in the prebiotic group including: *Bacteroides* (2w), *Flavonifactor* (2w), *Tyzzerella* (2w), and *Roseburia* (12w) (among others). Decreased relative abundance of genus *Blautia* at 12w was a feature shared by both placebo and prebiotic groups in males. Of note, at 12w the relative abundance of genus *Bifidobacterium* was decreased in the placebo group, whereas it was increased in the prebiotic group (Supplementary Figures 5C, D). Evaluation of SCFA-producing and Gram-negative genera demonstrated intervention-induced effects. Analysis of SCFA-producing bacteria revealed a significant time x intervention interaction (time: *p* = 0.065, intervention, *p* = 0.097, interaction: *p* = 0.026). *Post hoc* analysis revealed (1) the relative abundance of SCFA-producing bacteria increased in the prebiotic group at 2w and 12w compared to baseline and (2) the relative abundance of SCFA-producing bacteria was higher in the prebiotic group compared to the placebo group at 12w (Supplementary Figure 5E). Analysis of the Gram-negative taxa revealed a significant time x intervention interaction (time: *p* = 0.078, intervention, *p* = 0.120, interaction: *p* = 0.008) with *post hoc* analysis indicating that Gram-negative, proinflammatory bacteria were significantly decreased at 2w and 12w compared to baseline in the prebiotic group (Supplementary Figure 5F). Microbiota alpha diversity, beta diversity, and the curated relative abundances of SCFA-producing and Gram-negative-producing genera data are provided in Supplementary Data Sheets 2–5.

## Discussion

**FIGURE 7:**
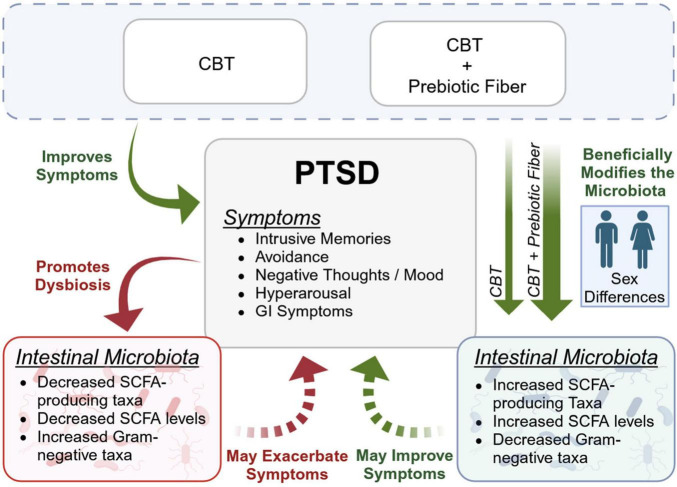
The interplay of cognitive behavioral therapy (CBT), prebiotic fiber, and the intestinal microbiome in post-traumatic stress disorder (PTSD). This figure highlights the impact of combined CBT and prebiotic fiber supplementation on the gut microbiome in individuals with PTSD. PTSD is often linked to a pro-inflammatory intestinal microbiome (i.e., dysbiosis) which could worsen symptoms and targeting the microbiome could be a strategy to complement existing treatment approaches. This study revealed that: (1) prebiotic fiber enhances the effects of CBT on the intestinal microbiome, (2) males show greater sensitivity to the prebiotic intervention than females, and (3) modulating the microbiome may improve PTSD symptoms in some individuals.

Accumulating evidence indicates that the intestinal microbiota may play an important role in psychiatric conditions like depression and anxiety as well as PTSD. Prior reports by our group and others demonstrate that PTSD is associated with stool microbiota dysbiosis and lower levels of serum SCFA compared to controls (Voigt et al., 2022; Bajaj et al., 2019; Hemmings et al., 2017; Malan-Muller et al., 2022; Levert-Levitt et al., 2022). The current proof-of-concept, pilot trial builds upon this literature to demonstrate: (1) that individuals with higher PCL-5 scores have subtle aberrations in the intestinal microenvironment and (2) a microbiota-modifying prebiotic intervention may positively impact outcomes in a subset of individuals with PTSD (Figure 7). These findings could be a road map to develop much needed new or adjunct therapies for individuals with PTSD.

In our prior report we found that veterans with PTSD who had low levels of SCFA also had higher levels of lipopolysaccharide binding protein (LBP), a marker of intestinal barrier dysfunction (Voigt et al., 2022). SCFA are anti-inflammatory and strengthen the intestinal barrier (Ney et al., 2023; Yao et al., 2022; Liu et al., 2021). It is plausible that increasing levels of SCFA could improve the intestinal barrier and directly and indirectly reduce systemic and neuroinflammation. This was the rationale to administer prebiotic fiber intervention to veterans with PTSD as an adjunct therapy to CPT in the current study. Although there was not a significant linear relationship between PCL-5 scores and stool SCFA there were differences in the abundance of some bacterial taxa including those capable of producing the SCFA propionate. The stool SCFA assessment in this study was a snapshot in time which reflects the abundance of bacteria capable of producing SCFA as well as dietary fiber consumption and time since consumption of the last meal. Future studies providing a standardized fiber containing meal and assessing blood levels of SCFA over time or *ex vivo* stool fermentation with fiber substrates are needed to understand the capability of PTSD patient microbiota to produce SCFA.

Analysis of the relationship between baseline PCL-5 score and stool microbiota revealed that three genera and a group of taxa capable of producing the SCFA propionate were negatively associated with PTSD severity. Interestingly, *Monoglobus*, Oscillospiraceae Unclassified, and Ruminococcaceae Unclassified were less abundant in participants with higher PCL-5 scores. These genera are members of the phyla Bacillota (formerly known as Firmicutes), class Clostridia with either documented or potential to produce SCFA (Kim et al., 2019; Gophna et al., 2017). These associations were weak (i.e., accounting for between 8 and 13% of PCL-5 score variability) and were no longer significant when correcting for multiple comparisons (i.e., *q* > 0.05), but these findings could be meaningful and future studies should investigate these associations further. Previous publications indicate that individuals with PTSD have a distinct microbiome and intestinal milieu from individuals without PTSD (Hemmings et al., 2017; Bajaj et al., 2019; Malan-Muller et al., 2022; Yoo et al., 2023; Zeamer et al., 2023; Levert-Levitt et al., 2022; Voigt et al., 2022) but there may also be aberrations that reflect PTSD severity, particularly in the abundance of SCFA-producing bacteria.

As a group, PCL-5 scores were not impacted by the microbiota-modifying prebiotic intervention, although beneficial changes were observed in the intestinal micro-environment. The prebiotic intervention was associated with higher relative abundance of SCFA-producing bacteria and reduced relative abundance of Gram-negative, proinflammatory bacteria (i.e., pathobionts) as would be expected for a prebiotic intervention and similar to what our group observed in Parkinson’s disease patients (Hall et al., 2023). There were similar taxonomic changes in the microbiota composition of both the placebo and prebiotic groups including reduced *Blautia* and reduced Lachnospiraceae and Oscillospiraceae families. Time was a significant factor for SCFA levels although the prebiotic intervention was associated with a transient increase in SCFA production at 2w. The common changes observed in both placebo and prebiotic groups may be driven by CPT [i.e., reductions in perceived stress (Tan, 2023)] or could reflect consumption of common components found in both the placebo and prebiotic bar, and will require further investigation.

A recent meta-analysis found that microbiota modulation is more effective at reducing depression symptoms in studies that have higher male enrollment (Zhang et al., 2023). Indeed, in the current study, male participants appeared to be more responsive to the prebiotic intervention than females. This effect was subtle, but *post hoc* analysis showed that significant symptom reduction at 12w was observed only when CPT was combined with prebiotic intervention. Why males were more sensitive to the effects of the prebiotic is not clear, but between sex differences were noted in microbiota composition at baseline, and males and females had unique microbiota responses to the prebiotic intervention. For example, while no significant prebiotic-induced changes were noted in the relative abundance of SCFA-producing or Gram-negative bacterial genera in females, the prebiotic intervention robustly increased the relative abundance of SCFA-producing bacterial genera and decreased Gram-negative bacterial genera in males. When examining specific bacterial genera, it was surprising that females in the placebo group had very few alterations in taxa, despite having significant reduction in PTSD symptoms at both 2w and 12w. One would expect reduced perceived stress to be associated with changes in the microbiota composition and it would be interesting to integrate objective measures of stress like cortisol in future studies. One difference worth noting between males in the placebo and prebiotic groups was the purported beneficial bacterium genus *Bifidobacterium* which had decreased relative abundance in the placebo group and increased relative abundance in the prebiotic group at 2w and 12w. Studies in rodents have demonstrated that administration of specific species of *Bifidobacterium* (e.g., *Bifidobacterium longum*, *Bifidobacterium infantis*) can ameliorate anxiety and depression-like behaviors and influence receptor expression and neurochemistry in the brain (Bercik et al., 2011; Bravo et al., 2011; Savignac et al., 2015; Desbonnet et al., 2008). While the changes in *Bifidobacterium* likely did not drive the clinical outcomes in the current study, it is intriguing to consider that loss of a beneficial bacteria in the placebo group at 12w could be a bellwether indicating failure to maintain long-term symptom reduction. The prebiotic intervention did not robustly impact stool SCFA levels in females or males which was surprising given the increased relative abundance of bacterial genera capable of producing SCFA. Shotgun metagenomic sequencing to annotate taxon abundances at the species level and gene/functional pathway evaluations could aid in reconciling these perhaps contradictory findings. It is important to note that this study assessed stool SCFA levels, and it would be of value to evaluate levels of SCFA in the systemic circulation in future investigations.

Consumption of prebiotic fiber can sometimes induce untoward side effects like bloating and gas. In the current study, participants did not self-report negative side effects of the prebiotic intervention in the post study questionnaire, important for a potential therapeutic. However, dropouts in the placebo and prebiotic groups reported side effects such as pain/cramping and bloating suggesting that fiber content in the bar (rather than the prebiotic fiber itself) may induce undesirable gastrointestinal symptoms in some individuals. PTSD is often associated with abdominal pain, diarrhea, constipation, bloating and nausea (Menon et al., 2013; Kearney et al., 2022). Importantly, significant beneficial changes in gastrointestinal function were observed during the study including reduced upper and lower abdominal pain, constipation, and nausea which were observed in both the placebo and prebiotic groups. This finding is significant as CPT appears to have beneficial effect on gastrointestinal symptoms in patients with PTSD in addition to the psychological benefits. Whether these changes reflect a reduction in PTSD symptoms, dietary changes associated with CPT, or components of the placebo or prebiotic intervention are unknown and will require additional investigations. It is important to note that the prebiotic intervention was associated with an additional benefit for some gastrointestinal outcomes including reduced diarrhea, cramping, and heartburn.

There are limitations associated with this proof-of-concept, pilot study. First, PTSD symptom severity was self-reported. Second, attrition occurred throughout the study; however, it is important to note that attrition is also noted as problematic for CPT with drop out estimates ranging from 35 to 40% (Schottenbauer et al., 2008; Goetter et al., 2015; Kehle-Forbes et al., 2016). The attrition resulted in fewer data points and samples to analyze at the 12w time point and may have contributed to a lack of power. Future studies with larger samples sizes are required to confirm the findings in this study. Third, only a subset of participants completed the post-study questionnaires. While the reported compliance was over 80% across both groups, if the participants who did not complete the post study questionnaires were largely non-compliant then this may have masked more robust treatment-induced effects as could participants over-reporting compliance. Fourth, microbiota communities were evaluated by 16S rRNA sequencing analysis and only stool SCFA were assessed. Future studies should utilize metagenomic sequencing to allow for identification of specific bacterial species/strains and systemic SCFA levels should be evaluated to develop a comprehensive understanding of how prebiotic intervention influenced levels of SCFA. Fifth, the SCFA and microbiota results at 2w are challenging to interpret, as participants consumed a non-habitual diet during this period (i.e., Blue Plate). Lastly, the prebiotic intervention used in this study was designed to specifically target microbiota aberrations present in patients with Parkinson’s disease and although both conditions are characterized by an increased relative abundance of proinflammatory bacteria and reduction in SCFA-producing bacteria, these microbiota profiles are not identical. Using different prebiotic fibers (including those targeted to the microbiota features observed in PTSD) could yield more robust treatment-induced effects. Similarly, it would be intriguing to utilize a symbiotic approach wherein both a probiotic (such as a *Bifidobacterium* species/strain) could be combined with the prebiotic intervention to maximize the potential benefit. The limitations associated with this proof-of-concept, pilot trial provide a roadmap for designing more robust microbiota-directed interventions for PTSD in the future.

In conclusion, there is an urgent need for new or adjunct treatment approaches for PTSD, particularly for veterans with PTSD who are treatment resistant (Murphy and Smith, 2018). Outcomes from this study suggest that a microbiota-directed intervention could have a meaningful impact on PTSD symptoms both psychological and gastrointestinal, but additional investigations are needed to fully understand this relationship.

## Data Availability

Sequencing reads generated in this study have been deposited in the National Center for Biotechnology Information (NCBI) BioProject database under accession numbers PRJNA1086950 (16S rRNA). The SILVA 16S rRNA database used for alignment is available at: https://data.qiime2.org/2021.11/common/silva-138-99-nb-classifier.qza.
