# Supplementary material for: Prebiotics as an adjunct therapy for posttraumatic stress disorder: a pilot randomized controlled trial
Source: Front Neurosci. 2025 Jan 7;18:1477519. doi: 10.3389/fnins.2024.1477519 (PMC11747240; doi:10.3389/fnins.2024.1477519)
Supplement: Supplementary file 6 [file Data_Sheet_6.docx]

Supplementary Material

# 1. Supplementary Data

- Supplementary Data Sheets uploaded individually as a Microsoft Excel file.

# 2. Supplementary Tables

- Supplemental Table 1: Post Bar Feedback Questionnaire.
- Supplemental Table 2: Linear Regression Analysis (PCL-5 with Taxa).
- Supplemental Table 3: Demographics table by sex (male, female) and intervention (placebo, prebiotic).
- Supplemental Table 4: Post Study Feedback.
- Supplemental Table 5: PROMIS GI results

## 3. Supplementary Figures

- Supplemental Figure 1: Significant between sex differences in microbiota composition at baseline.
- Supplemental Figure 2: The prebiotic intervention was not associated with significant changes in stool short chain fatty acids (SCFA) levels in males or females.
- Supplemental Figure 3: The prebiotic intervention does not influence alpha diversity.
- Supplemental Figure 4: The prebiotic intervention associated with taxonomic differences in stool microbiome community in females.
- Supplemental Figure 5: The prebiotic intervention associated with taxonomic differences in stool microbiota community based in males.

| **Supplementary Table 1: Post Bar Feedback Questionnaire** | |
| --- | --- |
| Question 1 | Total number of bars consumed during the study (out of 161) |
| Question 2 | How much did you like the study supplement overall? |
| Question 3 | Were you satisfied with the taste of the supplement? |
| Question 4 | Were you satisfied with the texture of the supplement? |
| Question 5 | Were you satisfied with the portion size of the supplement? |
| Question 6 | Would you want more of the supplement? |
| Question 7 | How much did the study supplement decrease your appetite during the day? |
| Question 8 | How easy was it to consume one bar per day? |
| Question 9 | How easy was it to consume two bars per day? |
| Question 10 | Given your experience, how easy would it be to consume three bars per day? |
| Question 11 | What is the likelihood that you would continue taking the study supplement? |
| 0 = Not at all, 5 = Moderately, 10 = Very much | |

| **Supplementary Table 2: Linear Regression Between PCL-5 and Stool Microbiota at Baseline** | | | | | | |
| --- | --- | --- | --- | --- | --- | --- |
|  | **Taxa Name** | ***R*^2^** | **F** | **p** | **q** | **β** |
| 1 | Total SCFA-Producing Taxa | <0.001 | F_(1,51)_=0.001 | 0.977 | 0.994 | 0.006 |
| 2 | Acetate-Producing Taxa | 0.012 | F_(1,51)_=0.627 | 0.432 | 0.798 | -0.060 |
| 3 | Butyrate-Producing Taxa | 0.004 | F_(1,51)_=0.196 | 0.660 | 0.851 | -0.049 |
| 4 | Propionate-Producing Taxa | 0.134 | F_(1,38)_=5.896 | **0.020** | 0.667 | -0.054 |
| 5 | Total Gram-Negative Taxa | 0.007 | F_(1,51)_=0.368 | 0.547 | 0.798 | 0.150 |
| 6 | Bacteroidota; Bacteroidia; Bacteroidales; Bacteroidaceae; Bacteroides | 0.007 | F_(1,51)_=0.347 | 0.558 | 0.798 | 0.146 |
| 7 | Bacteroidota; Bacteroidia; Bacteroidales; Prevotellaceae; Prevotella | 0.012 | F_(1,36)_=0.454 | 0.505 | 0.798 | 0.002 |
| 8 | Bacillota; Clostridia; Lachnospirales; Lachnospiraceae; Blautia | 0.014 | F_(1,50)_=0.717 | 0.401 | 0.798 | -0.040 |
| 9 | Bacillota; Clostridia; Oscillospirales; Ruminococcaceae; Faecalibacterium | 0.014 | F_(1,47)_=0.666 | 0.419 | 0.798 | 0.036 |
| 10 | Bacillota; Clostridia; Lachnospirales; Lachnospiraceae; Unclassified | 0.015 | F_(1,51)_=0.758 | 0.388 | 0.798 | -0.021 |
| 11 | Bacteroidota; Bacteroidia; Bacteroidales; Tannerellaceae; Parabacteroides | 0.067 | F_(1,51)_=3.679 | 0.061 | 0.667 | -0.045 |
| 12 | Bacillota; Clostridia; Lachnospirales; Lachnospiraceae; Agathobacter | 0.047 | F_(1,51)_=2.535 | 0.118 | 0.798 | -0.041 |
| 13 | Bacteroidota; Bacteroidia; Bacteroidales; Rikenellaceae; Alistipes | 0.013 | F_(1,48)_=0.638 | 0.429 | 0.798 | -0.014 |
| 14 | Bacillota; Clostridia; Oscillospirales; Ruminococcaceae; Subdoligranulum | 0.049 | F_(1,49)_=2.499 | 0.120 | 0.798 | -0.017 |
| 15 | Bacillota; Clostridia; Lachnospirales; Lachnospiraceae; Roseburia | 0.075 | F_(1,49)_=3.960 | 0.052 | 0.667 | -0.024 |
| 16 | Bacillota; Clostridia; Oscillospirales; Oscillospiraceae; UCG.002 | 0.001 | F_(1,47)_=0.029 | 0.865 | 0.958 | -0.002 |
| 17 | Bacillota; Negativicutes; Acidaminococcales; Acidaminococcaceae; Phascolarctobacterium | 0.006 | F_(1,44)_=0.259 | 0.613 | 0.826 | -0.004 |
| 18 | Proteobacteria; Gammaproteobacteria; Burkholderiales; Sutterellaceae; Sutterella | 0.016 | F_(1,43)_=0.696 | 0.409 | 0.798 | -0.007 |
| 19 | Bacillota; Clostridia; Lachnospirales; Lachnospiraceae; Lachnoclostridium | 0.022 | F_(1,47)_=1.054 | 0.310 | 0.798 | -0.007 |
| 20 | Bacillota; Clostridia; Lachnospirales; Lachnospiraceae; Fusicatenibacter | 0.024 | F_(1,51)_=1.254 | 0.268 | 0.798 | -0.013 |
| 21 | Verrucomicrobiota; Verrucomicrobiae; Verrucomicrobiales; Akkermansiaceae; Akkermansia | 0.036 | F_(1,39)_=1.440 | 0.237 | 0.798 | -0.001 |
| 22 | Bacillota; Clostridia; Oscillospirales; [Eubacterium] coprostanoligenes group; [Eubacterium] coprostanoligenes group | 0.031 | F_(1,39)_=1.251 | 0.270 | 0.798 | -0.002 |
| 23 | Actinobacteriota; Actinobacteria; Bifidobacteriales; Bifidobacteriaceae; Bifidobacterium | 0.004 | F_(1,44)_=0.196 | 0.660 | 0.851 | 0.002 |
| 24 | Bacillota; Clostridia; Oscillospirales; Ruminococcaceae; Unclassified | 0.083 | F_(1,45)_=4.098 | **0.049** | 0.667 | -0.007 |
| 25 | Bacillota; Clostridia; Lachnospirales; Lachnospiraceae; Lachnospira | <0.001 | F_(1,45)_=0.017 | 0.896 | 0.962 | -0.001 |
| 26 | Bacillota; Clostridia; Lachnospirales; Lachnospiraceae [Eubacterium] hallii group | 0.024 | F_(1,50)_=1.205 | 0.278 | 0.798 | -0.008 |
| 27 | Bacillota; Clostridia; Oscillospirales; Ruminococcaceae; Ruminococcus | 0.040 | F_(1,47)_=1.955 | 0.169 | 0.798 | -0.007 |
| 28 | Bacillota; Clostridia; Oscillospirales; Ruminococcaceae; [Eubacterium] siraeum group | 0.001 | F_(1,36)_=0.033 | 0.858 | 0.958 | <0.001 |
| 29 | Actinobacteriota; Coriobacteriia; Coriobacteriales; Coriobacteriaceae; Collinsella | 0.011 | F_(1,42)_=0.448 | 0.507 | 0.798 | 0.002 |
| 30 | Proteobacteria; Gammaproteobacteria; Enterobacterales; Enterobacteriaceae; Escherichia Shigella | 0.029 | F_(1,41)_=1.233 | 0.273 | 0.798 | -0.001 |
| 31 | Bacteroidota; Bacteroidia; Bacteroidales; Marinifilaceae; Odoribacter | <0.001 | F_(1,47)_=0.019 | 0.892 | 0.962 | <-0.001 |
| 32 | Bacillota; Clostridia; Lachnospirales; Lachnospiraceae; [Ruminococcus] torques group | 0.019 | F_(1,48)_=0.926 | 0.341 | 0.798 | -0.007 |
| 33 | Bacillota; Clostridia; Oscillospirales; Oscillospiraceae; UCG.005 | 0.020 | F_(1,36)_=0.743 | 0.394 | 0.798 | -0.001 |
| 34 | Bacillota; Clostridia; Lachnospirales; Lachnospiraceae; Lachnospiraceae  NK4A136 group | 0.071 | F_(1,43)_=3.271 | 0.078 | 0.670 | -0.007 |
| 35 | Bacillota; Clostridia; Christensenellales; Christensenellaceae;  Christensenellaceae R.7 group | 0.004 | F_(1,43)_=0.162 | 0.689 | 0.859 | 0.001 |
| 36 | Bacillota; Clostridia; Lachnospirales; Lachnospiraceae; Anaerostipes | <0.001 | F_(1,48)_=0.007 | 0.936 | 0.994 | <-0.001 |
| 37 | Proteobacteria; Gammaproteobacteria; Burkholderiales; Sutterellaceae; Parasutterella | 0.042 | F_(1,36)_=1.573 | 0.218 | 0.798 | -0.002 |
| 38 | Bacillota; Clostridia; Lachnospirales; Lachnospiraceae; Dorea | 0.005 | F_(1,50)_=0.268 | 0.607 | 0.826 | -0.003 |
| 39 | Bacillota; Negativicutes; Acidaminococcales; Acidaminococcaceae;  Acidaminococcus | 0.030 | F_(1,38)_=1.171 | 0.286 | 0.798 | 0.001 |
| 40 | Bacillota; Bacilli; Lactobacillales; Streptococcaceae; Streptococcus | 0.011 | F_(1,43)_=0.479 | 0.493 | 0.798 | -0.002 |
| 41 | Bacillota; Clostridia; Oscillospirales; Oscillospiraceae; NK4A214 group | 0.008 | F_(1,44)_=0.375 | 0.543 | 0.798 | -0.003 |
| 42 | Bacillota; Clostridia; Lachnospirales; Lachnospiraceae; Coprococcus | <0.001 | F_(1,45)_<0.001 | 0.984 | 0.994 | <0.001 |
| 43 | Bacteroidota; Bacteroidia; Bacteroidales; Prevotellaceae; Paraprevotella | 0.038 | F_(1,42)_=1.631 | 0.209 | 0.798 | -0.002 |
| 44 | Bacillota; Negativicutes; Veillonellales-Selenomonadales; Veillonellaceae;  Dialister | <0.001 | F_(1,45)_=0.001 | 0.970 | 0.994 | <-0.001 |
| 45 | Bacillota; Clostridia; Lachnospirales; Lachnospiraceae; Lachnospiraceae  UCG-004 | 0.015 | F_(1,44)_=0.677 | 0.415 | 0.798 | -0.002 |
| 46 | Bacillota; Clostridia; Peptostreptococcales-Tissierellales; Peptostreptococcaceae; Romboutsia | 0.032 | F_(1,42)_=1.366 | 0.249 | 0.798 | -0.001 |
| 47 | Bacillota; Clostridia; Oscillospirales; Ruminococcaceae; Incertae Sedis | 0.001 | F_(1,48)_=0.033 | 0.857 | 0.958 | <-0.001 |
| 48 | Bacillota; Clostridia; Lachnospirales; Lachnospiraceae; [Eubacterium] eligens group | 0.046 | F_(1,41)_=1.982 | 0.167 | 0.798 | -0.003 |
| 49 | Bacillota; Clostridia; Oscillospirales; Butyricicoccaceae; Butyricicoccus | 0.001 | F_(1,50)_=0.063 | 0.803 | 0.951 | 0.001 |
| 50 | Bacillota; Bacilli; Erysipelotrichales; Erysipelotrichaceae; Holdemanella | 0.008 | F_(1,35)_=0.276 | 0.603 | 0.826 | <0.001 |
| 51 | Bacillota; Clostridia; Lachnospirales; Lachnospiraceae; Tyzzerella | 0.008 | F_(1,46)_=0.349 | 0.557 | 0.798 | -0.001 |
| 52 | Bacillota; Clostridia; Oscillospirales; Oscillospiraceae; UCG-003 | 0.010 | F_(1,49)_=0.496 | 0.485 | 0.798 | -0.002 |
| 53 | Bacillota; Clostridia; Lachnospirales; Lachnospiraceae; [Ruminococcus]  gauvreauii group | 0.028 | F_(1,47)_=1.374 | 0.247 | 0.798 | 0.003 |
| 54 | Bacillota; Clostridia; Oscillospirales; Oscillospiraceae; Oscillibacter | 0.008 | F_(1,46)_=0.368 | 0.547 | 0.798 | -0.001 |
| 55 | Bacillota; Clostridia; Clostridiales; Clostridiaceae; Clostridium sensu stricto 1 | 0.018 | F_(1,40)_=0.730 | 0.398 | 0.798 | 0.001 |
| 56 | Desulfobacterota; Desulfovibrionia; Desulfovibrionales; Desulfovibrionaceae;  Bilophila | 0.001 | F_(1,49)_=0.032 | 0.858 | 0.958 | <0.001 |
| 57 | Bacillota; Clostridia; Oscillospirales; Oscillospiraceae; Colidextribacter | 0.009 | F_(1,50)_=0.452 | 0.505 | 0.798 | -0.001 |
| 58 | Bacillota; Clostridia; Monoglobales; Monoglobaceae; Monoglobus | 0.083 | F_(1,46)_=4.140 | **0.048** | 0.667 | -0.004 |
| 59 | Bacillota; Clostridia; Lachnospirales; Lachnospiraceae; [Ruminococcus] gnavus group | 0.004 | F_(1,45)_=0.185 | 0.669 | 0.851 | -0.001 |
| 60 | Bacillota; Bacilli; Erysipelotrichales; Erysipelatoclostridiaceae;  Erysipelotrichaceae UCG-003 | 0.008 | F_(1,46)_=0.382 | 0.539 | 0.798 | -0.001 |
| 61 | Bacillota; Clostridia; Oscillospirales; Oscillospiraceae; Flavonifractor | 0.018 | F_(1,48)_=0.879 | 0.353 | 0.798 | 0.001 |
| 62 | Bacillota; Clostridia; Clostridia vadinBB60 group; Clostridia vadinBB60 group; Clostridia vadinBB60 group | 0.074 | F_(1,44)_=3.503 | 0.068 | 0.667 | <0.001 |
| 63 | Bacillota; Clostridia; Oscillospirales; Oscillospiraceae; Unclassified | 0.099 | F_(1,48)_=5.280 | **0.026** | 0.667 | -0.003 |
| 64 | Bacillota; Clostridia; Lachnospirales; Lachnospiraceae; Lachnospiraceae ND3007 group | 0.021 | F_(1,43)_=0.923 | 0.342 | 0.667 | 0.001 |
| 65 | Bacillota; Clostridia; Lachnospirales; Lachnospiraceae; CAG.56 | 0.003 | F_(1,40)_=0.103 | 0.750 | 0.904 | <0.001 |
| 66 | Bacillota; Clostridia; Oscillospirales; Ruminococcaceae; Negativibacillus | 0.009 | F_(1,40)_=0.366 | 0.548 | 0.798 | <-0.001 |
| 67 | Bacillota; Clostridia; Oscillospirales; Oscillospiraceae; Uncultured | 0.003 | F_(1,46)_=0.150 | 0.700 | 0.859 | <-0.001 |
| 68 | Bacteroidota; Bacteroidia; Bacteroidales; Marinifilaceae; Butyricimonas | 0.009 | F_(1,48)_=0.436 | 0.512 | 0.798 | -0.001 |
| Note: Firmicutes were renamed Bacillota in 2023. | | | | | | |

| **Supplementary Table 3: Demographics Table by sex and intervention (Figure 3 B-C)** | | | | |
| --- | --- | --- | --- | --- |
|  | **Male** | | **Female** | |
|  | **Placebo** | **Prebiotic** | **Placebo** | **Prebiotic** |
| **N=** | 16 | 16 | 19 | 19 |
| **Age (mean ± SEM, range)** | 48.4 ± 2.7 (31-67) | 41.9 ± 2.3 (31-61) | 44.8 ± 2.6 (25-67) | 42.1 ± 2.1 (28-62) |
| ***Sex***  Female (n,%)  Male (n,%) | 0 (0.0%)  16 (100.0%) | 0 (0.0%)  16 (100.0%) | 19 (100.0%)  0 (0.0%) | 19 (100.0%)  0 (0.0%) |
| ***Race***  Asian  Black or African American  Native Hawaiian or Pacific Islander  Other  White | 2 (12.5%)  5 (31.3%)  0 (0.0%)  0 (0.0%)  9 (56.3%) | 1 (6.3%)  0 (0.0%)  1 (6.3%)  1 (6.3%)  13 (81.3%) | 1 (5.3%)  4 (21.1%)  0 (0.0%)  3 (15.8%)  11 (57.9%) | 0 (0.0%)  3 (15.8%)  0 (0.0%)  2 (10.5%)  14 (73.7%) |
| ***Ethnicity***  Not Hispanic or Latino  Hispanic or Latino | 14 (87.5%)  2 (12.5%) | 15 (93.8%)  1 (6.3%) | 14 (73.7%)  5 (26.3%) | 16 (84.2%)  3 (15.8%) |
| ***Military Service Status***  Active Duty  Discharged  Inactive Ready Reserve  Medically Retired  Reserves  Retired  Not Indicated | 3 (18.8%)  5 (31.3%)  0 (0.0%)  4 (25.0%)  0 (0.0%)  3 (18.8%)  1 (6.3%) | 1 (6.3%)  9 (56.3%)  1 (6.3%)  3 (18.8%)  2 (12.5%)  0 (0.0%)  0 (0.0%) | 1 (5.3%)  12 (63.2%)  0 (0.0%)  1 (5.3%)  1 (5.3%)  3 (15.8%)  1 (5.3%) | 4 (21.1%)  10 (52.6%)  0 (0.0%)  2 (10.5%)  1 (5.3%)  2 (10.5%)  0 (0.0%) |
| ***Cohort Type***  Combat  Military Sexual Trauma | 13 (81.3%)  3 (18.8%) | 14 (87.5%)  2 (12.5%) | 5 (26.3%)  14 (73.7%) | 6 (31.6%)  13 (68.4%) |
| ***PCL-5 (0-80)***  Baseline  2w  12w | 56.4 ± 2.8 (n=16)  35.6 ± 4.9 (n=16)  44.9 ± 8.3 (n=8) | 59.9 ± 4.3 (n=16)  30.7 ± 4.3 (n=15)  38.1 ± 5.5 (n=11) | 56.4 ± 2.2 (n=19)  39.3 ± 4.3 (n=19)  37.1 ± 4.8 (n=14) | 52.8 ± 2.6 (n=19)  35.4 ± 3.8 (n=19)  38.8 ± 3.5 (n=15) |

| **Supplementary Table 4: Post Study Questionnaires (completed at Week 12). Mean ± standard error of the mean (SEM), range, median** | | | | |
| --- | --- | --- | --- | --- |
|  |  | **Whole Cohort (n=70)** | **Placebo**  **(n=35)** | **Prebiotic**  **(n=35)** |
| **Feasibility and Acceptability**  **(0 = not at all, 5 = moderately, 10 = very easy / very much)** | | | | |
| **Compliance** | Response Rate | N=44 (63%) | N=23 (66%) | N=21 (60%) |
|  | Total Bars Consumed (total 161) | 138 ± 5  (40-161) (n=32)  Median = 148 | 129 ± 8  (40-161) (n=18)  Median = 142 | 151 ± 3.5  (122-161) (n=15)  Median = 160 |
| **Feasibility** | How easy was it to consume  *one bar per day*? | 8.07 ± 0.41  (0-10) (n=44)  Median = 10 | 8.26 ± 0.51  (5-10) (n=23)  Median = 10 | 7.86 ± 0.65  (0-10) (n=21)  Median = 10 |
|  | How easy was it to consume  *two bars per day*? | 5.34 ± 0.60  (0-10) (n=44)  Median = 5 | 5.87 ± 0.81  (0-10) (n=23)  Median = 5 | 4.76 ± 0.88  (0-10) (n=21)  Median = 5 |
|  | Given your experience, how easy would it be to consume *three bars per day*? | 3.75 ± 0.63  (0-10) (n=44)  Median = 2.5 | 3.91 ± 0.83  (0-10) (n=23)  Median = 5 | 3.57 ± 0.98  (0-10) (n=21)  Median = 0 |
| **Acceptability** | How much did you *like* the study supplement overall? | 6.25 ± 0.49  (0-10) (n=44)  Median = 5 | 6.09 ± 0.70  (0-10) (n=23)  Median = 5 | 6.43 ± 0.70  (0-10) (n=21)  Median = 5 |
|  | How much did the study supplement *decrease your appetite* during the day? | 3.52 ± 0.48  (0-10) (n=44)  Median = 5 | 3.70 ± 0.65  (0-10) (n=23)  Median = 5 | 3.33 ± 0.72  (0-10) (n=21)  Median = 5 |
|  | Were you satisfied with the *taste* of the supplement? | 5.57 ± 0.49  (0-10) (n=44)  Median = 5 | 5.00 ± 0.70  (0-10) (n=23)  Median = 5 | 6.19 ± 0.68  (0-10) (n=21)  Median = 5 |
|  | Were you satisfied with the *texture* of the supplement? | 6.25 ± 0.49  (0-10) (n=44)  Median = 5 | 5.87 ± 0.75  (0-10) (n=23)  Median = 5 | 6.67 ± 0.63  (0-10) (n=21)  Median = 5 |
|  | Were you satisfied with the *portion size* of the supplement? | 6.74 ± 0.47  (0-10) (n=43)  Median = 5 | 7.17 ± 0.61  (0-10) (n=23)  Median = 5 | 6.25 ± 0.71  (0-10) (n=20)  Median = 5 |
|  | What is the likelihood that you would *continue taking* the intervention? | 6.48 ± 0.62  (0-10) (n=44)  Median = 10 | 5.87 ± 0.92  (0-10) (n=23)  Median = 5 | 7.14 ± 0.81  (0-10) (n=21)  Median = 10 |

| **Supplementary Table 5: PROMIS GI Results** | | | | | | | | |
| --- | --- | --- | --- | --- | --- | --- | --- | --- |
|  |  | **Placebo** | | | **Prebiotic** | | | **Mixed Effect Analysis** |
|  |  | **BL (n=26)** | **2w (n=26)** | **12w (n=23)** | **BL (n=28)** | **2w (n=21)** | **12w (n=21)** |  |
| **Gas** | Bloating | 4.77±0.54 | 4.88±0.69 | 3.65±0.53 | 5.54±0.62 | 4.00±0.52 | 3.33±0.42 | **T: *p<0.01*,** I: p=0.72, TxI: p=0.13 |
|  | Belching | 3.81±0.56 | 3.44±0.64 | 3.04±0.47 | 4.14±0.56 | 3.24±0.61 | 2.76±0.50 | **T: *p<0.01*,** I: p=0.70, TxI: p=0.47 |
|  | Passing Gas | 5.62±0.63 | 5.44±0.66 | 3.87±0.45 | 5.61±0.63 | 4.29±0.49 | 4.67±0.64 | **T: *p=0.03*,** I: p=0.95, TxI: p=0.15 |
|  | Excessive Gas Overall | 5.46±0.72 | 4.31±0.74 | 3.83±0.54 | 4.93±0.67 | 3.81±0.55 | 4.14±0.73 | **T: *p=0.03***, I: p=0.94, TxI: p=0.46 |
| **Upper GI Problems** | Upper Abdominal Pain | 3.58±0.49 | 3.38±0.59 | 2.65±0.52 | 3.43±0.49 | 2.33±0.55 | 2.24±0.40 | **T: *p<0.01***, I: p=0.34, TxI: p=0.37 |
|  | Upper Abdominal Cramping | 2.65±0.38 | 2.94±0.64 | 2.00±0.36 | 2.64±0.46 | 2.05±0.50 | 1.81±0.29 | **T: *p=0.01***, I: p=0.30, TxI: p=0.15 |
|  | Indigestion | 3.96±0.60 | 3.69±0.62 | 2.74±0.47 | 4.46±0.56 | 2.62±0.55 | 2.38±0.39 | **T: *p<0.01***, I: p=0.39, TxI: p=0.07 |
|  | Heartburn | 4.04±0.64 | 3.44±0.76 | 2.74±0.50 | 4.75±0.66 | 2.29±0.59 | 2.05±0.35 | **T: *p<0.01***, I: p=0.67, **TxI: *p=0.04*** |
|  | Acid Taste in Mouth | 2.84±0.46 | 2.44±0.56 | 1.96±0.27 | 3.14±0.59 | 1.75±0.53 | 1.86±0.43 | **T: *p=0.01***, I: p=0.69, TxI: p=0.43 |
|  | Difficulty Swallowing | 2.00±0.35 | 1.63±0.26 | 1.65±0.32 | 1.75±0.34 | 1.71±0.38 | 1.48±0.25 | T: p=0.10, I: p=0.50, TxI: p=0.99 |
|  | Food Coming up to Mouth | 2.54±0.52 | 1.56±0.30 | 2.09±0.36 | 2.96±0.54 | 1.76±0.48 | 1.86±0.42 | **T: *p=0.02***, I: p=0.90, TxI: p=0.63 |
|  | Nausea | 2.96±0.36 | 2.56±0.57 | 2.48±0.53 | 4.00±0.57 | 2.76±0.65 | 2.14±0.39 | **T: *p<0.01***, I: p=0.86, TxI: p=0.06 |
|  | Nausea with Eating | 2.54±0.41 | 2.38±0.48 | 1.78±0.38 | 3.04±0.55 | 2.71±0.62 | 1.76±0.28 | **T: *p=0.01***, I: p=0.90, TxI: p=0.50 |
|  | Vomiting | 1.85±0.33 | 1.94±0.60 | 1.44±0.19 | 2.43±0.47 | 1.76±0.49 | 1.52±0.21 | T: p=0.07, I: p=0.72, TxI: p=0.46 |
|  | Less Food Intake with Symptoms | 3.08±0.56 | 2.44±0.55 | 2.26±0.49 | 3.64±0.68 | 2.52±0.64 | 2.10±0.40 | **T: *p=0.01***, I: p=0.85, TxI: p=0.64 |
|  | Pain with Eating | 2.31±0.34 | 2.25±0.64 | 1.52±0.26 | 2.93±0.53 | 2.52±0.63 | 1.57±0.25 | **T: *p<0.01***, I: p=0.61, TxI: p=0.50 |
|  | Pain with Swallowing | 1.42±0.25 | 1.19±0.19 | 1.39±0.31 | 1.61±0.33 | 1.43±0.34 | 1.29±0.16 | T: p=0.47, I: p=0.89, TxI: p=0.71 |
|  | Weight Loss | 2.46±0.48 | 1.88±0.46 | 1.87±0.38 | 2.54±0.48 | 2.71±0.67 | 2.05±0.42 | T: p=0.14, I: p=0.61, TxI: p=0.69 |
|  | Loss of Appetite | 3.85±0.48 | 2.75±0.53 | 2.30±0.40 | 3.82±0.65 | 3.57±0.70 | 2.57±0.53 | **T: *p<0.01***, I: p=0.88, TxI: p=0.86 |
|  | Intolerance to Foods | 3.46±0.68 | 3.31±0.92 | 2.22±0.49 | 2.71±0.47 | 2.10±0.52 | 1.52±0.27 | **T: *p=0.01***, I: p=0.13, TxI: p=0.46 |
| L**ower GI Problems** | Lower Abdominal Pain | 3.73±0.54 | 3.69 ±0.66 | 3.26±0.55 | 4.07±0.48 | 3.24±0.54 | 2.76±0.53 | **T: *p=0.03***, I: p=0.47, TxI: p=0.16 |
|  | Lower Abdominal Cramping | 3.00±0.47 | 3.13±0.77 | 2.78±0.44 | 3.86±0.48 | 2.67±0.50 | 2.67±0.50 | **T: *p<0.05***, I: p=0.92, **TxI: *p=0.04*** |
|  | Alternating BMs | 4.42±0.74 | 4.38±0.88 | 3.26±0.69 | 3.36±0.50 | 2.48±0.52 | 2.52±0.34 | T: p=0.06, I: p=0.08, TxI: p=0.41 |
|  | Constipated >70% | 4.27±0.67 | 4.06±0.72 | 2.52±0.52 | 3.61±0.70 | 2.67±0.56 | 2.62±0.52 | **T: *p<0.01***, I: p=0.50, TxI: p=0.10 |
|  | Hard Stools | 3.69±0.56 | 4.81±0.75 | 2.83±0.42 | 4.86±0.57 | 3.52±0.62 | 3.24±0.50 | **T: *p=0.01***, I: p=0.48, **TxI: *p=0.02*** |
|  | Straining w/ BMs | 3.96±0.51 | 4.50±0.85 | 2.65±0.50 | 4.93±0.63 | 3.95±0.70 | 3.14±0.58 | **T: *p<0.01***, I: p=0.45, TxI: p=0.14 |
|  | Sensation of Incomplete Bowel Emptying | 4.62±0.72 | 4.13±0.80 | 3.70±0.73 | 5.11±0.70 | 3.52±0.66 | 3.43±0.68 | **T: *p<0.01***, I: p=0.95, TxI: p=0.48 |
|  | Diarrhea > 70% | 3.39±0.63 | 3.06±0.69 | 2.87±0.64 | 2.36±0.39 | 2.00±0.45 | 1.57±0.21 | T: p=0.16, **I: *p=0.04***, TxI: p=0.30 |
|  | Irregular Bowel Habits | 4.81±0.67 | 5.00±0.92 | 3.57±0.67 | 4.29±0.63 | 4.00±0.71 | 3.14±0.59 | **T: *p=0.01***, I: p=0.47, TxI: p=0.40 |
|  | Infrequent BMs | 3.69±0.71 | 4.75±0.96 | 2.91±0.65 | 3.96±0.67 | 4.10±0.77 | 3.00±0.58 | **T: *p=0.02***, I: p=0.71, TxI: p=0.70 |
|  | Watery Stools | 3.42±0.62 | 3.13±0.61 | 2.78±0.53 | 3.50±0.49 | 2.62±0.54 | 2.29±0.34 | **T: *p=0.03***, I: p=0.49, TxI: p=0.25 |
|  | Soft Stools | 4.77±0.64 | 5.94±0.73 | 3.96±0.61 | 4.75±0.58 | 4.38±0.54 | 3.48±0.48 | **T: *p=0.01***, I: p=0.28, **TxI: *p=0.02*** |
|  | Mucus in Stool | 3.15±0.64 | 2.88±0.85 | 2.26±0.53 | 2.43±0.52 | 2.19±0.50 | 2.14±0.51 | T: p=0.11, I: p=0.51, TxI: p=0.30 |
|  | Blood in Stool | 1.62±0.27 | 2.75±0.81 | 1.09±0.09 | 1.57±0.28 | 1.43±0.38 | 1.19±0.13 | **T: *p=0.02***, I: p=0.30, **TxI: *p=0.04*** |
|  | Frequent BMs | 4.81±0.62 | 4.81±0.82 | 3.04±0.52 | 3.93±0.59 | 3.62±0.67 | 3.48±0.64 | **T: *p=0.05***, I: p=0.36, TxI: p=0.12 |
|  | Fecal Urgency | 4.58±0.69 | 4.19±0.84 | 3.30±0.51 | 3.93±0.61 | 2.81±0.58 | 3.71±0.64 | T: p=0.12, I: p=0.56, TxI: p=0.21 |
|  | Fecal Incontinence | 1.96±0.44 | 2.81±0.82 | 2.09±0.44 | 2.36±0.47 | 1.57±0.38 | 2.05±0.48 | T: p=0.89, I: p=0.77, TxI: p=0.08 |
| T = Time, I = Intervention, TxI = Time x Intervention Interaction | | | | | | | | |


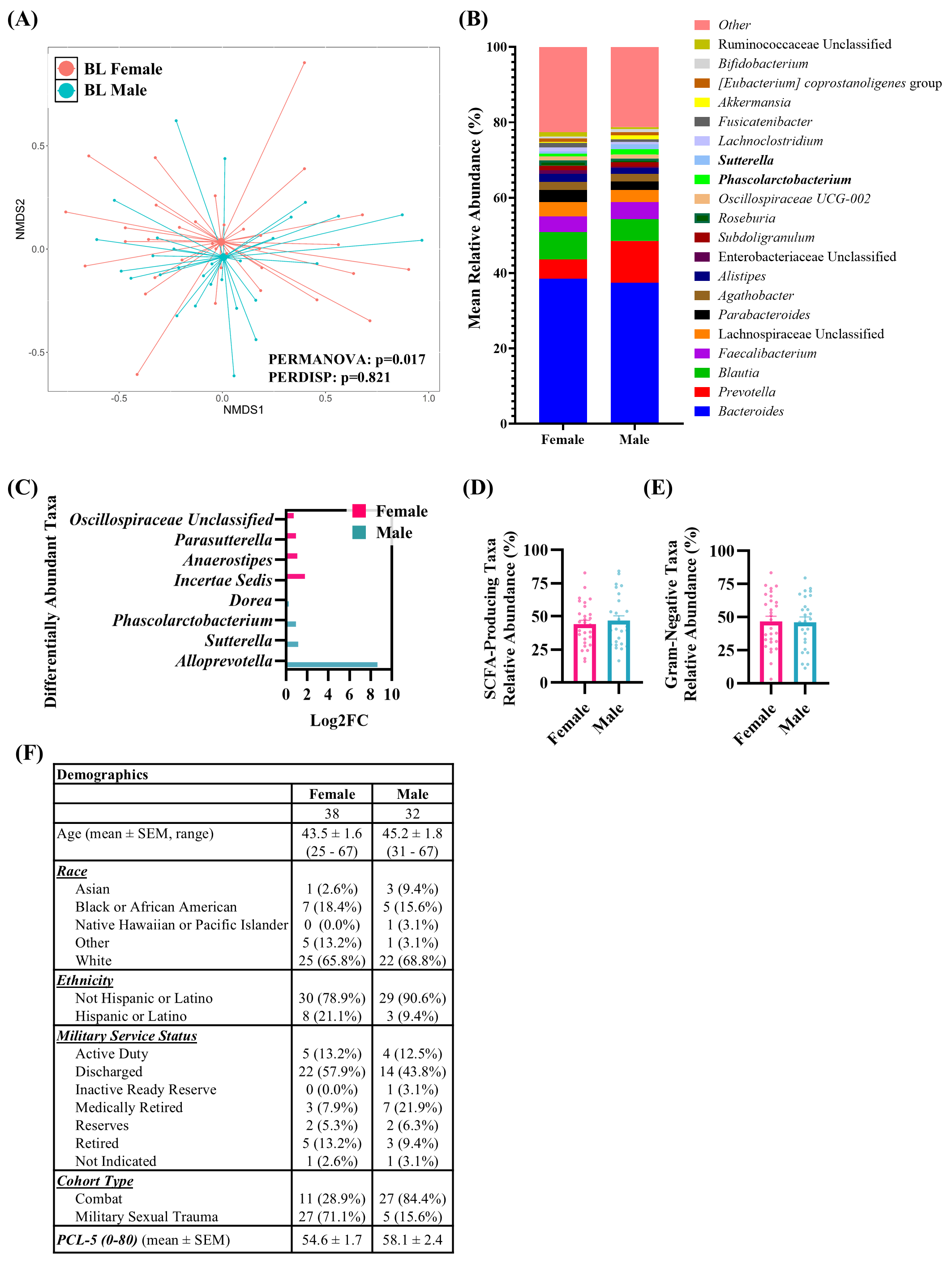


**Supplementary Figure 1.** **Significant between sex differences in microbiota composition at baseline.** **(A)** Significant differences in male and female microbiota communities were observed (PERMANOVA: p=0.017; PERMDISP: p = 0.821; Centroid based NMDS plot). **(B)** Stacked histograms show the mean relative abundances of microbial genera between sexes, corresponding with **(C)** differentially abundant genera log2 fold change values between sexes (CLR-KW: bold, q<0.05). **(D-E)** Analysis of curated lists of genera revealed no between sex differences in the relative abundances of SCFA-producing taxa or Gram-negative taxa (Student’s *t*-test: p > 0.05). **(F)** Demographics by sex. See **Supplementary Data Sheets 3, 4.**


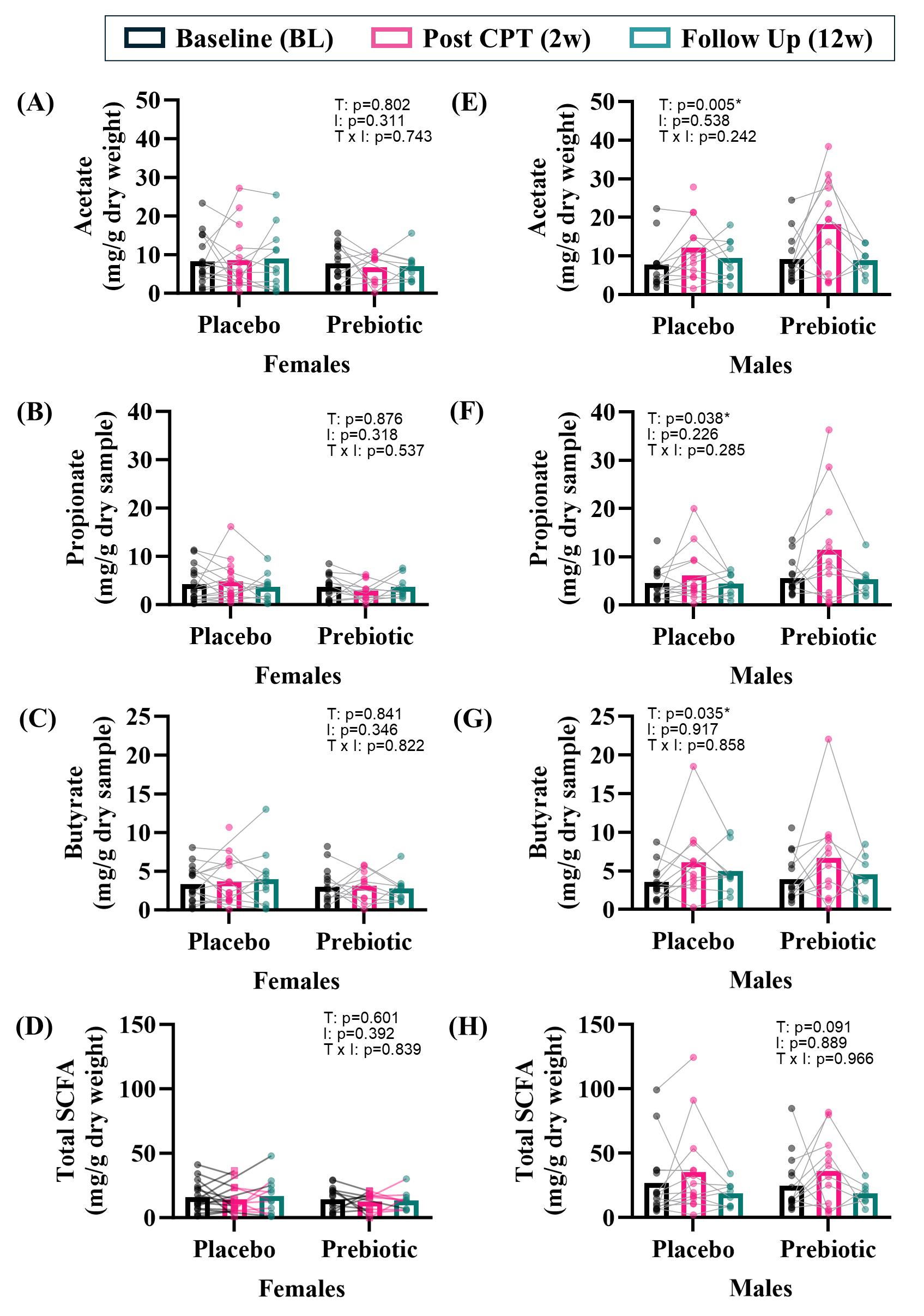


**Supplementary Figure 2: The prebiotic intervention was not associated with significant changes in stool short chain fatty acids (SCFA) levels in males or females.** Females: No significant main effects or *post hoc* effects were observed: **(A)** Acetate (time: p=0.802, intervention: p=0.311, interaction: p=0.743), **(B)** Propionate (time: p=0.876, intervention: p=0.318, interaction: p=0.537), **(C)** Butyrate (time: p=0.841, intervention: p=0.346, interaction: p=0.822), **(D)** Total SCFA (i.e., acetate + propionate + butyrate; time: p=0.601, intervention: p=0.392, interaction: p=0.839), **(E)** Acetate (time: p=0.005, intervention: p=0.538, interaction: p=0.242), **(F)** Propionate (time: 0.038, intervention: p=0.226, interaction: p=0.285), **(G)** Butyrate (time: 0.035, intervention: p=0.917, interaction: p=0.858), **(H)** Total SCFA (time: 0.091, intervention: p=0.889, interaction: p=0.966). Outliers from each group were omitted prior to analysis. Females: Placebo: n=11-17/group; Prebiotic: n=9-17/group; Males: Placebo: n=9-15/group, Prebiotic: n=7-15/group. Two-way mixed model ANOVA (factors: time, intervention) with *post hoc* Tukey.


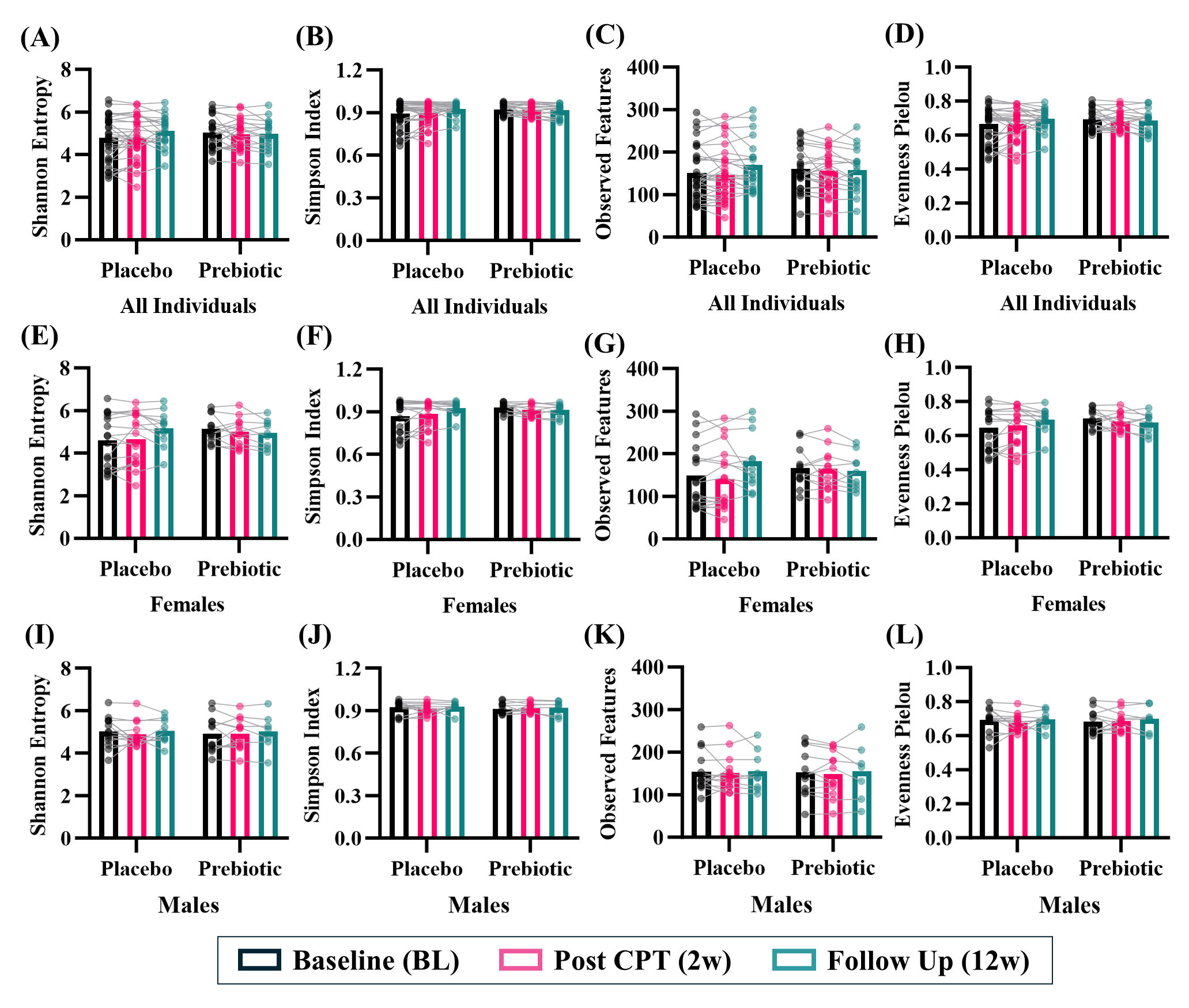


**Supplementary Figure 3: The prebiotic intervention was not associated with changes in alpha diversity.** Evaluation of Shannon Entropy, Simpson Index, Observed Features, and Evenness Pielou revealed no significant differences in **(A-D)** All individuals, **(E-H)** Females, or **(I-L)** Males. All individuals: Placebo: n=20-31, Prebiotic: n=16-24. Females: Placebo: n=11-17, Prebiotic: n=9-12. Males: Placebo: n=9-14, Prebiotic: n=7-12. Two-way mixed model ANOVA (factors: time, intervention) with *post hoc* Tukey.


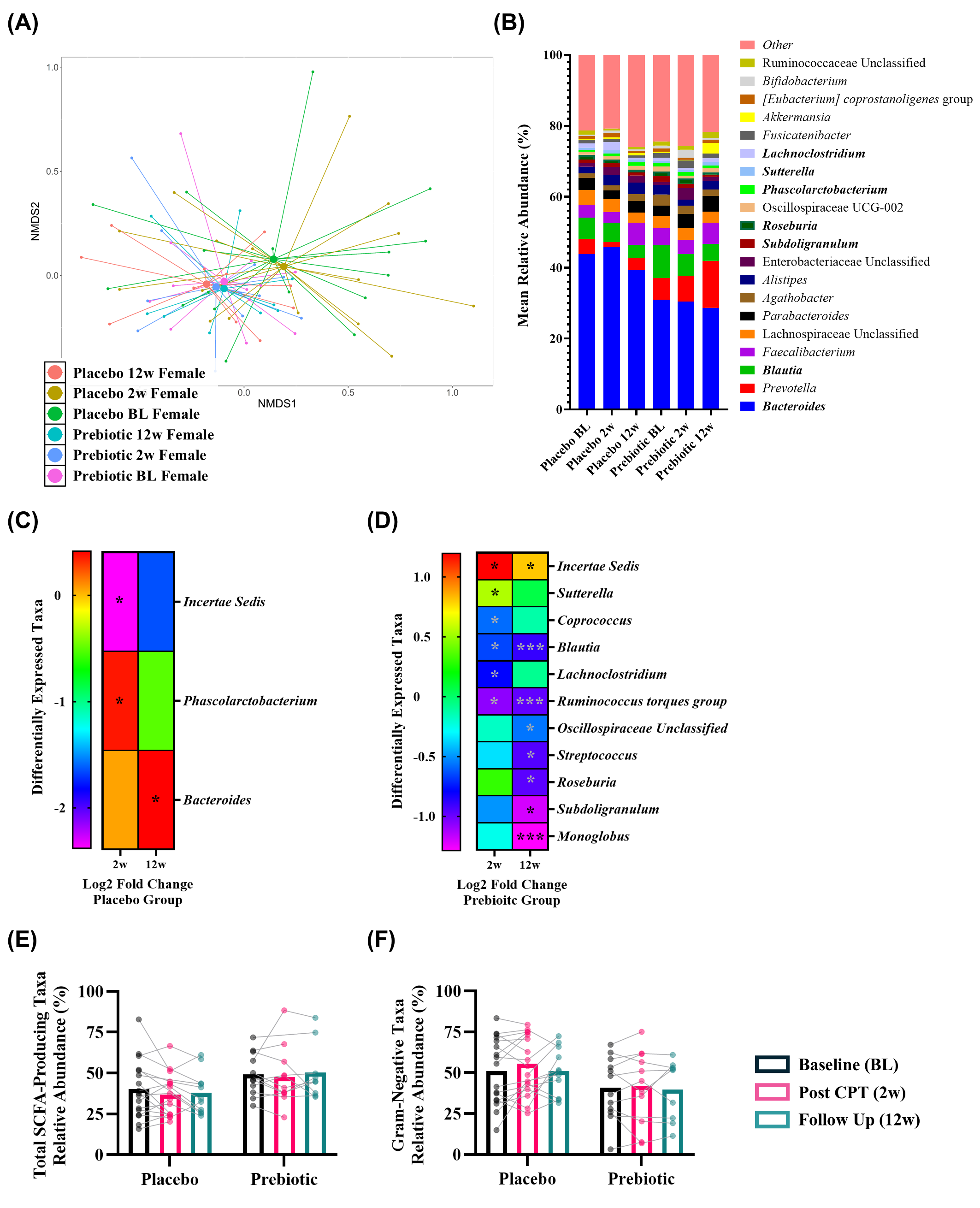


**Supplementary Figure 4: The prebiotic intervention associated with taxonomic differences in stool microbiome community in females:** **(A)** There were no between group differences in overall microbial community structure (PERMANOVA, p > 0.05). **(B)** Stacked histogram displays the mean relative abundance of microbial genera by group (Wilcoxon-signed rank test: bold, q < 0.001 (corrected for multiple comparisons)). Analysis of differentially abundant genera as log2 fold change from baseline revealed differences in specific genera in **(C)** placebo and **(D)** prebiotic groups (Wilcoxon signed-rank test: * q<0.05, ** q<0.01, *** q< 0.001 (corrected for multiple comparisons)). Analysis of curated lists of genera revealed **(E)** SCFA-Producing Taxa. There was a main effect of the intervention (time: p=0.254, intervention: p=0.025, interaction: p=0.252), but no *post hoc* differences were identified. **(F)** Gram-Negative, Proinflammatory Taxa. A significant main effect of the intervention was identified (time: p=0.164, intervention: p=0.044, interaction: p=0.170) but no *post hoc* differences were identified. (**A-B**) (PERMANOVA/PERDISP: **Supplementary Data Sheet 3**; Centroid based NMDS plot, Aitchinson distance). (**C-D**) Wilcoxon-signed rank test: **Supplementary Data Sheet 4**. (**E-F**) Mixed Model ANOVA with *post hoc* Tukey.


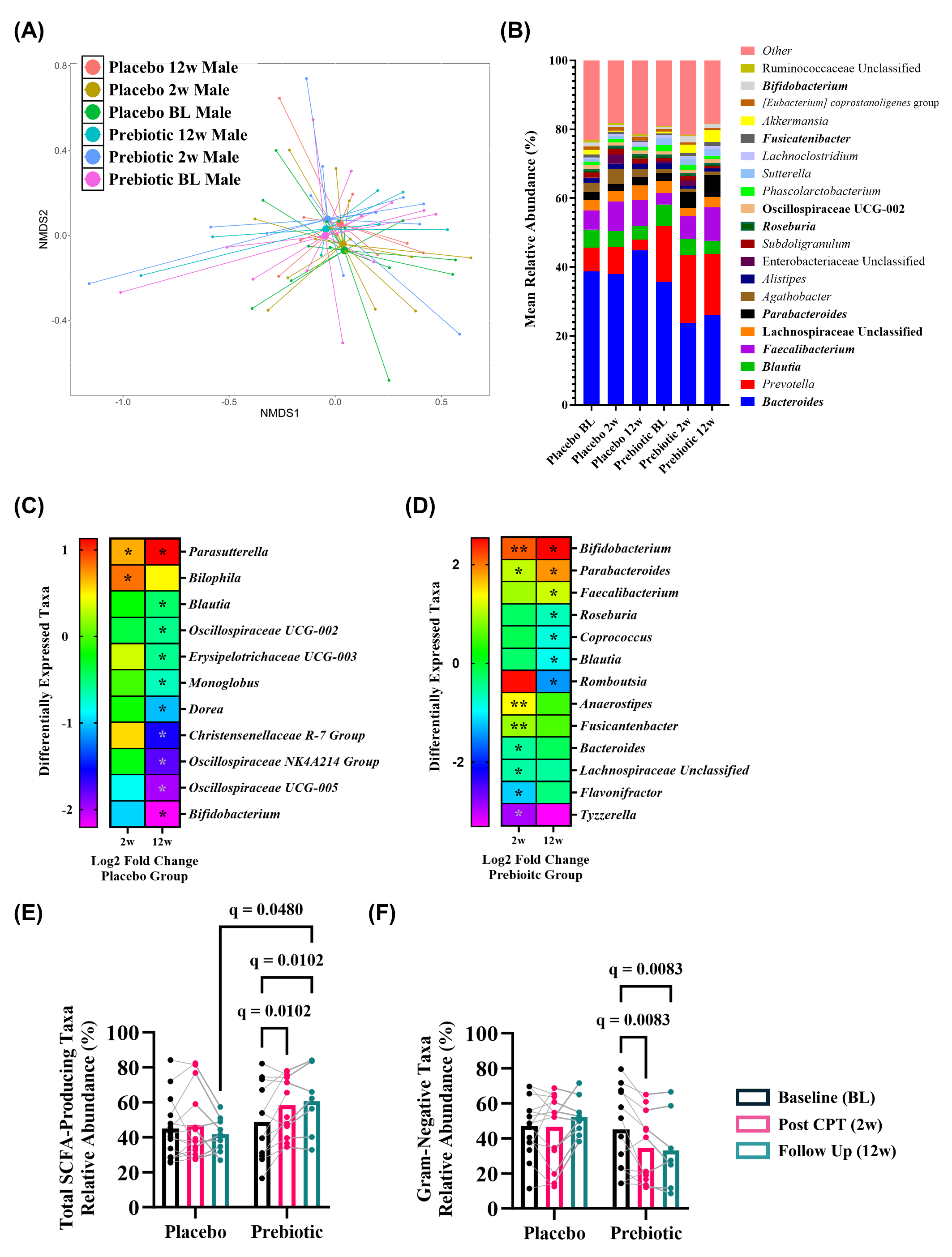


**Supplementary Figure 5: The prebiotic intervention associated with taxonomic differences in stool microbiota community based in males.** **(A)** There were no between group differences in overall microbial community structure (PERMANOVA, p > 0.05). **(B)** Stacked histograms demonstrate the mean relative abundance of microbial genera for each group across time (Wilcoxon-signed rank test: bold, q < 0.01 (corrected for multiple comparisons)). Analysis of differentially abundant genera as log2 fold change from baseline revealed differences in specific genera in **(C)** placebo and **(D)** prebiotic groups (Wilcoxon signed-rank test: * q<0.05, ** q<0.01 (corrected for multiple comparisons)). Analysis of curated lists of genera revealed: **(E)** SCFA-producing Taxa. There was a significant time x intervention interaction (time: p=0.065, intervention: p=0.097, interaction: p=0.026) and *post hoc* testing revealed the relative abundance of SCFA-producing taxa was higher in the prebiotic group compared to the placebo group at 12w and the prebiotic group had significantly increased SCFA-producing taxa at 2w compared to baseline. **(F)** Gram-negative, Proinflammatory Taxa. There was a significant time x intervention interaction (time: p=0.078, intervention: p=0.120, interaction: p=0.008) and *post hoc* testing indicated the relative abundance of Gram-negative taxa was significantly decreased in the prebiotic group (baseline vs 2w and 12w). (**A-B**) (PERMANOVA/PERDISP: **Supplementary Data Sheet 3**; Centroid based NMDS plot, Aitchinson distance). (**C-D**) Wilcoxon-signed rank test: **Supplementary Data Sheet 4**. (**E-F**) Mixed Model ANOVA with *post hoc* Tukey.
